# Relationships among changes in walking and sedentary behaviors, individual attributes, changes in work situation, and anxiety during the COVID-19 pandemic in Japan

**DOI:** 10.1016/j.pmedr.2021.101640

**Published:** 2021-11-15

**Authors:** Shohei Nagata, Hiroki M. Adachi, Tomoya Hanibuchi, Shiho Amagasa, Shigeru Inoue, Tomoki Nakaya

**Affiliations:** a Graduate School of Environmental Studies, Tohoku University, 468-1 Aoba, Aramaki, Aoba-ku, Sendai 980-8572, Japan; b Department of Preventive Medicine and Public Health, Tokyo Medical University, 6-1-1 Shinjuku, Shinjuku-ku, Tokyo 160-8402, Japan

**Keywords:** Physical activity, Walking, Sedentary behavior, Anxiety, COVID-19, Japan, **ADI**, Area deprivation index, CFI, Comparative fit index, DID, Densely-inhabited-district, NPIs, Non-pharmaceutical interventions, RMSEA, Root mean square error of approximation, SES, Socioeconomic status, SoE, State of emergency

## Abstract

Studies from many countries, including Japan, have reported decreased physical activity during the coronavirus disease 2019 (COVID-19) pandemic. However, the individual attributes as related to changes in physical activity during the pandemic in Japan have been scarcely investigated. The present study explored the relationships among individual attributes including demographic, socioeconomic, and geographic characteristics, work situation changes, perception of anxiety, and changes in walking and sedentary behaviors, during the pandemic in Japan. To obtain data indicating individual circumstances during the first wave of the pandemic in Japan, we conducted a nationwide online survey from May 19 to May 23, 2020 (n = 1,200). To observe changes in walking behavior objectively and retrospectively, we collected data on the number of daily steps as measured by the iPhone’s Health application. Path analysis was employed to examine relationships between individual attributes and changes in walking and sedentary behaviors. Decreased physical activity, especially, decreased walking behavior among younger individuals and those living in highest-density neighborhoods were identified. There was increased sedentary behavior among females. Moreover, individuals with higher socioeconomic status (SES) tended to become inactive due to work-from-home/standby-at-home and individuals with lower SES tended to become inactive due to decreased amount of work. Decreased walking behavior and increased sedentary behavior were associated with a perception of strong anxiety related to the pandemic. Our findings would be helpful in considering measures to counteract health risks during the pandemic by taking into account individual backgrounds.

## Highlights

• Females, younger people, those living in the highest-density tended to be inactive.
• Inactivity was related to standby/work-from-home for higher socioeconomic people.
• Inactivity was related to decreased amount of work for lower socioeconomic people.
• Decreased physical activity was related to strong anxiety during the pandemic.

## 1 Introduction

The novel coronavirus disease (COVID-19) pandemic is ongoing as of August 2021. Although vaccinations have been administered in many countries, the World Health Organization reported that as of August 3, 2021, the number of new COVID-19 cases per week worldwide is still increasing (World Health Organization, 2021). Up until August 10, 2021, more than 200 million cases have been confirmed globally, including more than 4.3 million deaths (Johns Hopkins Coronavirus Resource Center, 2021). To reduce the spread of infection, non-pharmaceutical interventions (NPIs), such as social distancing measures and lockdowns, were implemented in many countries (BBC, 2021, Courtemanche et al., 2020, Lau et al., 2020). In Japan, to prevent the health system from collapsing due to rapid increase in new positive cases, the government declared a state of emergency (SoE) four times until August 10, 2021 and requested people to show self-restraining behaviors such as shortening business hours at restaurants, avoiding nonessential outings, and working from home (Office for Novel Coronavirus Disease Control, Cabinet Secretariat, Government of Japan, 2020).

Previous studies have revealed the effects of changes in human mobility related to NPIs during the COVID-19 outbreak (Amagasa et al., 2020, Badr et al., 2020, Li et al., 2020, Nagata et al., 2021, Yabe et al., 2020, Zhou et al., 2020). Reduced mobility that effectively reduces the spread of infection could cause physical inactivity, which is concerning (Hall et al., 2021, Lippi et al., 2020). A descriptive study showed that globally, within 30 days of declaration of the pandemic, there was a 27.3% reduction in the mean number of steps taken (Tison et al., 2020). Further, several studies have shown that reduced physical activity during the COVID-19 pandemic is associated with mental health issues, such as depression, loneliness, stress, anxiety, and sadness (Meyer et al., 2020, Pieh et al., 2020, Silva et al., 2020). Additionally, according to the World Health Organization, physical inactivity is a major risk factor for non-communicable diseases (World Health Organization, 2020) and is estimated to cause 6–10% of coronary heart diseases, type 2 diabetes, and breast and colon cancers worldwide (Lee et al., 2012). Therefore, it is important to analyze reduced physical activity caused by the implementation of NPIs to plan long-term measures against the current and any future pandemics.

Much has been discussed on the factors encouraging or discouraging physical activity. Many studies consistently demonstrate that individuals of higher socioeconomic status (SES) are more likely to be physically active during leisure time, while those of lower SES are more likely to engage in job-related physical activity (Beenackers et al., 2012, McNeill et al., 2006). Furthermore, neighborhood environments are also associated with the amount of physical activity. High-walkability neighborhoods, which have mixed land use or favorable esthetic qualities, promote residents’ walking behavior (Saelens et al., 2003, Saelens and Handy, 2008, Sallis et al., 2009), but those living in highly-deprived neighborhoods are more likely to be physically inactive (Cubbin et al., 2006, Hillsdon et al., 2008). During the COVID-19 pandemic, people with low incomes in the USA and the UK showed decreased physical activity (Fearnbach et al., 2021, Robinson et al., 2021). Conversely, a study from Bangladesh demonstrated that highly educated individuals with high income is more likely to be physically inactive (Rahman et al., 2020). Considering such differences reflect social structures and the measures taken against COVID-19 in each country; further evaluation based on the situations in different countries is needed.

In Japan, decreased physical activity was observed during the COVID-19 outbreak (Hino and Asami, 2021, Makizako et al., 2021, Yamada et al., 2020). Hino and Asami (2021) suggested that proximity to large parks could effectively mitigate decreased walking among female older adults during the SoE. Hanibuchi et al. (2021) reported that reduced time spent outdoors is associated with individual attributes such as age, gender, income, or residential location and perception of anxiety related to the infection or the stigma. Moreover, Koohsari et al. (2021) revealed that the implementation of working-from-home was associated with decreased work-related physical activity and increased sitting time. However, few studies have attempted to clarify the comprehensive relationships between physical inactivity and individual attributes such as age, residential location, work situation changes, or anxiety related to the pandemic. The present study explored the relationships among individual attributes including demographic, socioeconomic, and geographic characteristics, work situation changes, perception of anxiety, and changes in physical activity, specifically decreased walking and increased sedentary behaviors, during the first wave of the COVID-19 pandemic in Japan.

## 2 Methods

### 2.1 Data collection

We conducted a nationwide online survey among registered panel members of a survey company (Cross Marketing Inc.) from May 19 to May 23, 2020. People aged 20–69 years with diverse demographic and socioeconomic backgrounds, owning iPhones, and living in Japan were recruited from 4.65 million panel members. The quota sample was designed to have the same distribution of population by age, gender, and geographical region based on the 2015 Japan population census. As for the definition of the geographical region, we classified the prefectures into metropolitan areas that consist of Tokyo, Kanagawa, Saitama, Chiba, Aichi, Gifu, Mie, Osaka, Hyogo, Kyoto and Nara and nonmetropolitan areas that consist of the rest. However, this sampling design was not applied to participants aged 60–69 years because of fewer responses from females of this age group.

### 2.2 Measurement of changes in walking and sedentary behaviors

To analyze changes in sedentary behavior, the participants were asked regarding change is duration of sitting since COVID-19 outbreak when compared with before the pandemic. Participants selected answers from the following options: significant reduction, slight reduction, no change, slight rise, significant rise. The answers were converted to integer values (1: significant reduction, 2: slight reduction, 3: no change, 4: slight rise, 5: significant rise) to obtain ordinal variables for statistical analyses.

Additionally, to observe changes in walking behavior objectively, from the participants, we collected screenshot images of the pre-installed “iPhone Health App” (Apple Inc.), which automatically records daily step counts. The screenshots of number of daily steps were captured for the previous 3 months by the participants. We then extracted this information through image processing and optical character recognition methods using Python 3.7.7, OpenCV 4.2.0, and Tesseract 5.0.0. Details of the image processing methods to obtain the step counts in numbers have been described previously (Adachi et al., 2021). Subsequently, we calculated the differences between the number of mean steps before and after the first SoE for each participant and used them for each period to measure the changes in walking behavior. According to a previous study, the pre-SoE period was from February 19, 2020 to March 23, 2020 and the post-SoE period was from April 16, 2020 to May 19, 2020 (Adachi et al., 2021).

### 2.3 Demographic, socioeconomic, and geographic variables

The demographic and socioeconomic attributes of the participants considered as variables were: gender (0: males, 1: females), age (20–29 years, 30–39 years, 40–49 years, 50–59 years, 60–69 years), chronic disease (0: no, 1: yes), educational status (junior high school/high school, junior (technical) college/vocational school, undergraduate/graduate school), occupation (white-collar job including administrators, professionals, and office clerks; gray-collar job including sales clerks and service workers; blue-collar job including security workers and production, construction, and transportation workers; and other/not working), household annual income (<3 million yen, 3–7 million yen, ≥7 million yen, and unknown), living alone (0: no, 1: yes), living with child(ren) under 18 years (0: no, 1: yes), and living with person(s) aged 65 years and older (0: no, 1: yes). The data corresponding to the categorical variables, such as age, educational status, occupation, and household income, were converted to a binary system indicating whether the participants belong to each group or not (0: no, 1: yes).

Neighborhood-level population density and deprivation were considered as geographic variables. The neighborhoods were defined by the postal code of the participants’ residential address and were categorized as follows into groups of approximately equal sample sizes: lowest density (non-densely-inhabited-district (non-DID), defined by the 2015 Japan population census), middle-low density (DID with 7,015 people/km^2^ or fewer), middle-high density (DID with 7,016–10,214 people/km^2^), and highest density (DID with 10,215 people/km^2^ or more). The neighborhood-level deprivation indicator was calculated using the area deprivation index (ADI) derived from the 2015 Japan population census, which has been explained previously (Nakaya et al., 2014). A higher ADI indicates that the neighborhood has more deprived conditions. We categorized the neighborhoods based on ADI quartiles as lowest ADI, middle-low ADI, middle-high ADI, and highest ADI groups.

### 2.4 Variables representing changes related to work situation and anxiety

We hypothesized that the work situation changes and perception of anxiety due to COVID-19 are also associated with walking and sedentary behaviors. The variables that indicate changes in work situation were: introduction of work-from-home/standby-at-home measures (0: no, 1: yes) and decreased amount of work (0: no, 1: yes). Three anxiety variables were also used: strong anxiety about getting infected (0: no, 1: yes), spreading the infection to others (0: no, 1: yes), and stigma associated with going out (0: no, 1: yes).

### 2.5 Statistical analysis

**Fig. 1:**
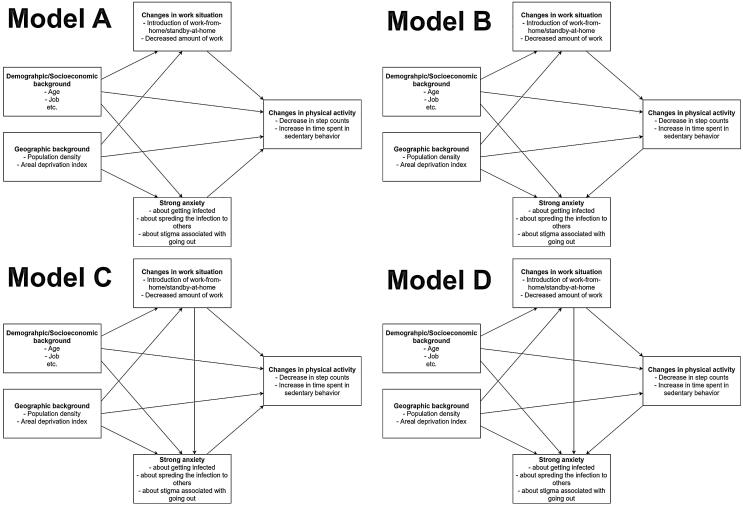
Conceptual frameworks of the models indicating hypotheses of the relationships among individual attributes, work situation changes, perception of anxiety, and changes in physical activity.

To examine the direct and indirect relationships among the individual attributes including demographic, socioeconomic, and geographic variables, changes in work situation, perception of anxiety due to the pandemic, and changes in physical activity, we assumed the following models (Fig. 1): model A represents that the individuals’ background affected their work situation and perception of anxiety which affected the changes in physical activity; model B assumes the inverse relationship between perception of anxiety and the changes in physical activity represented by model A to account for the possibility that the physical inactivity causes increased anxiety during the pandemic (Silva et al., 2020); model C and D assumes the direct relationship between the work situation changes and perception of anxiety in addition to the frameworks of model A and B. All models assume the direct relationships between the individuals’ background and the changes in physical activity.

We examined the relationships by path analysis, a special case of structural equation modeling, and evaluated the most suitable model to explain the comprehensive relationships by comparative fit index (CFI) and root mean square error of approximation (RMSEA) which indicate how well the model fits. CFI is expressed as a value between 0 and 1, and models with a value greater than 0.95 are often interpreted as good fitting (Ullman and Bentler, 2012). RMSEA represents that the smaller the value, the better fitting the model, and values of 0.06 or less can be interpreted as good fitting (Ullman and Bentler, 2012). Furthermore, to evaluate the relationship between each variable, the relationship was considered statistically significant if the path’s p-value was<0.05. To estimate the coefficients to the dichotomous variables indicating changes in work situation and perception of anxiety and ordinal variables indicating changes in time spent in sedentary behavior, binary probit and ordered probit regression models were employed, respectively. All statistical analyses were performed using R 3.6.1, and the lavaan package, version 0.6–7, was used to run path analysis.

This study was approved by the Research Ethics Committee of the Graduate School of Engineering, Tohoku University (approval number: 20A-3). Informed consent was obtained from all participants.

## 3 Results

**Fig. 2:**
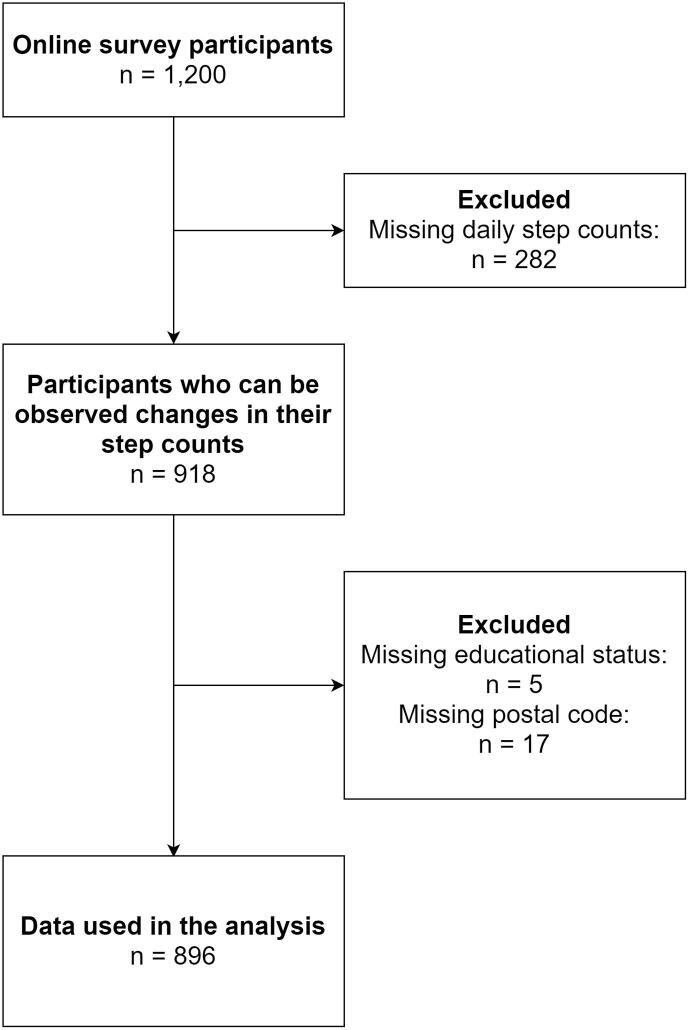
Flow diagram of participant inclusion.

In the online survey, 1,200 panel members participated. We excluded the data of 282 participants from whom the daily step counts could not be obtained for more than 15 days both before and after the SoE because of errors such as low image resolution. We excluded the data of five participants whose educational data was unavailable and 17 participants whose postal code was missing. Finally, data of 896 participants were used for analyses (Fig. 2).

**Fig. 3:**
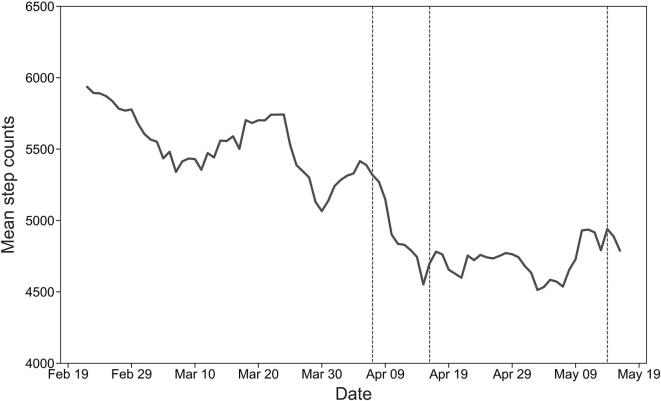
Changes in mean step counts of the participants (7-day moving average). Vertical dash lines represent the start/end date of state of emergency (SoE) declaration (April 7, 2020, SoE declaration for Tokyo, Osaka, Kanagawa, Saitama, Chiba, Hyogo, and Fukuoka; April 16, 2020, SoE declaration for the remaining prefectures; May 14, 2020, lifting of SoE for prefectures excluding Hokkaido, Saitama, Chiba, Tokyo, Kanagawa, Kyoto, Osaka, and Hyogo).

Fig. 3 shows the differences in mean step counts between late-February and mid-May 2020. The step counts decreased gradually from late-February, and there was more than 20% reduction after the SoE. Table 1 summarizes the changes in walking and sedentary behaviors during the COVID-19 outbreak based on demographic, socioeconomic, and geographic attributes. More than 60% of participants increasingly spent time in sedentary behavior. The average step count consistently decreased across all attributes, while sedentary behavior increased during the outbreak. There were significant differences in step count reductions when considering age, educational status, living with child(ren), neighborhood density, ADI, and introduction of work-from-home/standby-at-home measures. Furthermore, the changes in time spent in sedentary behavior across groups categorized according to gender, educational status, neighborhood density, ADI, introduction of work-from-home/standby-at-home, decreased amount of work, and strong anxiety about getting infected, spreading the infection to others, and the stigma associated with going out, were significant.

**Table 1:** Changes in walking and sedentary behaviors during the COVID-19 outbreak based on characteristics of respondents.

	**n**	**%**	**Average change in step counts ^a)b)^**	**Number of participants by change levels of sedentary behavior ^c)^**					
				Significant reduction	Slight reduction	No change	Slight rise	Significant rise	
Total	896		−918.4 (SD = 2276.2)	1 (0.1%)	9 (1.0%)	321 (35.8%)	293 (32.7%)	272 (30.4%)	
Gender			*p = 0.263*	*p = 0.002*					
Males	459	51.2	−1001.5 (SD = 2318.7)	1 (0.2%)	5 (1.1%)	178 (38.8%)	160 (34.9%)	115 (25.1%)	
Females	437	48.8	−831.2 (SD = 2230.0)	0 (0.0%)	4 (0.9%)	143 (32.7%)	133 (30.4%)	157 (35.9%)	
Age			*p < 0.001*	*p = 0.066*					
20–29 years	134	15.0	−1991.5 (SD = 2604.2)	0 (0.0%)	3 (2.2%)	40 (29.9%)	34 (25.4%)	57 (42.5%)	
30–39 years	180	20.1	−771.0 (SD = 2586.5)	0 (0.0%)	2 (1.1%)	60 (33.3%)	64 (35.6%)	54 (30.0%)	
40–49 years	214	23.9	−907.6 (SD = 1903.6)	0 (0.0%)	1 (0.5%)	78 (36.4%)	71 (33.2%)	64 (29.9%)	
50–59 years	171	19.1	−756.1 (SD = 1921.4)	1 (0.6%)	1 (0.6%)	73 (42.7%)	52 (30.4%)	44 (25.7%)	
60–69 years	197	22.0	−475.9 (SD = 2186.2)	0 (0.0%)	2 (1.0%)	70 (35.5%)	72 (36.5%)	53 (26.9%)	
Chronic disease			*p = 0.388*	*p = 0.983*					
No	682	76.1	−952.9 (SD = 2338.1)	1 (0.1%)	7 (1.0%)	243 (35.6%)	225 (33.0%)	206 (30.2%)	
Yes	214	23.9	−808.5 (SD = 2067.8)	0 (0.0%)	2 (0.9%)	78 (36.4%)	68 (31.8%)	66 (30.8%)	
Educational status			*p < 0.001*	*p = 0.025*					
Junior high school/high school	177	19.8	−493.9 (SD = 1849.1)	1 (0.6%)	1 (0.6%)	77 (43.5%)	52 (29.4%)	46 (26.0%)	
Junior (technical) college/vocational school	219	24.4	−668.6 (SD = 2506.6)	0 (0.0%)	3 (1.4%)	84 (38.4%)	69 (31.5%)	63 (28.8%)	
Undergraduate/graduate school	500	55.8	−1178.1 (SD = 2276.6)	0 (0.0%)	5 (1.0%)	160 (32.0%)	172 (34.4%)	163 (32.6%)	
Occupation			*p = 0.788*	*p = 0.601*					
White-collar job	407	45.4	−901.1 (SD = 1876.6)	0 (0.0%)	3 (0.7%)	152 (37.3%)	126 (31.0%)	126 (31.0%)	
Gray-collar job	152	17.0	−1067.1 (SD = 2910.4)	0 (0.0%)	1 (0.7%)	57 (37.5%)	45 (29.6%)	49 (32.2%)	
Blue-collar job	75	8.4	−913.1 (SD = 2575.5)	0 (0.0%)	0 (0.0%)	31 (41.3%)	27 (36.0%)	17 (22.7%)	
Other/not working	262	29.2	−860.6 (SD = 2343.2)	1 (0.4%)	5 (1.9%)	81 (30.9%)	95 (36.3%)	80 (30.5%)	
Household annual income			*p=0.347*	*p=0.085*					
Less than 3 million yen	122	13.6	−808.0 (SD=3125.0)	0 (0.0%)	3 (2.5%)	50 (41.0%)	35 (28.7%)	34 (27.9%)	
3–7 million yen	377	42.1	−886.4 (SD=2082.5)	0 (0.0%)	2 (0.5%)	128 (34.0%)	121 (32.1%)	126 (33.4%)	
7 million yen or more	298	33.3	−950.5 (SD=1965.7)	1 (0.3%)	2 (0.7%)	100 (33.6%)	108 (36.2%)	87 (29.2%)	
Unknown	99	11.0	−1079.9 (SD=2619.3)	0 (0.0%)	2 (2.0%)	43 (43.4%)	29 (29.3%)	25 (25.3%)	
Living alone			*p=0.134*	*p=0.363*					
No	736	82.1	−856.5 (SD=2159.7)	1 (0.1%)	6 (0.8%)	267 (36.3%)	246 (33.4%)	216 (29.3%)	
Yes	160	17.9	−1203.5 (SD=2739.1)	0 (0.0%)	3 (1.9%)	54 (33.8%)	47 (29.4%)	56 (35.0%)	
Living with child(ren) under 18 years			*p<0.001*	*p=0.226*					
No	650	72.5	−1084.2 (SD=2295.6)	1 (0.2%)	7 (1.1%)	225 (34.6%)	213 (32.8%)	204 (31.4%)	
Yes	246	27.5	−480.3 (SD=2168.3)	0 (0.0%)	2 (0.8%)	96 (39.0%)	80 (32.5%)	68 (27.6%)	
Living with person(s) aged 65 years and older			*p=0.423*	*p=0.091*					
No	762	85.0	−942.0 (SD=2314.5)	1 (0.1%)	6 (0.8%)	268 (35.2%)	248 (32.5%)	239 (31.4%)	
Yes	134	15.0	−784.7 (SD=2047.5)	0 (0.0%)	3 (2.2%)	53 (39.6%)	45 (33.6%)	33 (24.6%)	
Neighborhood density			*p<0.001*	*p<0.001*					
Lowest density	218	24.3	−360.0 (SD=2117.8)	1 (0.5%)	1 (0.5%)	99 (45.4%)	66 (30.3%)	51 (23.4%)	
Middle-low density	232	25.9	−506.8 (SD=2046.2)	0 (0.0%)	0 (0.0%)	87 (37.5%)	80 (34.5%)	65 (28.0%)	
Middle-high density	229	25.6	−1029.4 (SD=2268.8)	0 (0.0%)	3 (1.3%)	81 (35.4%)	80 (34.9%)	65 (28.4%)	
Highest density	217	24.2	−1802.4 (SD=2399.2)	0 (0.0%)	5 (2.3%)	54 (24.9%)	67 (30.9%)	91 (41.9%)	
Areal deprivation index (ADI)			*p=0.002*	*p=0.019*					
Lowest ADI	224	25.0	−1118.2 (SD=2699.2)	1 (0.4%)	3 (1.3%)	75 (33.5%)	66 (29.5%)	79 (35.3%)	
Middle-low ADI	224	25.0	−1278.3 (SD=2359.6)	0 (0.0%)	0 (0.0%)	66 (29.5%)	84 (37.5%)	74 (33.0%)	
Middle-high ADI	224	25.0	−661.9 (SD=2055.4)	0 (0.0%)	2 (0.9%)	93 (41.5%)	71 (31.7%)	58 (25.9%)	
Highest ADI	224	25.0	−615.3 (SD=1840.5)	0 (0.0%)	4 (1.8%)	87 (38.8%)	72 (32.1%)	61 (27.2%)	
Introduction of work-from-home/standby-at-home			*p<0.001*	*p<0.001*					
No	595	66.4	−638.5 (SD=1996.7)	1 (0.2%)	5 (0.8%)	249 (41.8%)	193 (32.4%)	147 (24.7%)	
Yes	301	33.6	−1471.8 (SD=2664.6)	0 (0.0%)	4 (1.3%)	72 (23.9%)	100 (33.2%)	125 (41.5%)	
Decreased amount of work			*p=0.080*	*p<0.001*					
No	724	80.8	−849.8 (SD=2234.5)	1 (0.1%)	7 (1.0%)	284 (39.2%)	233 (32.2%)	199 (27.5%)	
Yes	172	19.2	−1207.2 (SD=2429.5)	0 (0.0%)	2 (1.2%)	37 (21.5%)	60 (34.9%)	73 (42.4%)	
Strong anxiety about getting infected			*p=0.806*	*p=0.002*					
No	613	68.4	−932.1 (SD=2112.0)	1 (0.2%)	6 (1.0%)	233 (38.0%)	209 (34.1%)	164 (26.8%)	
Yes	283	31.6	−888.8 (SD=2600.3)	0 (0.0%)	3 (1.1%)	88 (31.1%)	84 (29.7%)	108 (38.2%)	
Strong anxiety about spreading the infection to others			*p=0.499*	*p=0.002*					
No	668	74.6	−883.5 (SD=2063.6)	1 (0.1%)	6 (0.9%)	253 (37.9%)	226 (33.8%)	182 (27.2%)	
Yes	228	25.4	−1020.8 (SD=2810.7)	0 (0.0%)	3 (1.3%)	68 (29.8%)	67 (29.4%)	90 (39.5%)	
Strong anxiety about stigma associated with going out			*p=0.942*	*p<0.001*					
No	745	83.1	−915.2 (SD=2070.3)	1 (0.1%)	7 (0.9%)	280 (37.6%)	251 (33.7%)	206 (27.7%)	
Yes	151	16.9	−934.4 (SD=3107.0)	0 (0.0%)	2 (1.3%)	41 (27.2%)	42 (27.8%)	66 (43.7%)	

^a)^ Changes in step counts were calculated by the differences in the mean step counts between the pre-SoE period (from February 19, 2020 to March 23, 2020) the post-SoE period (from April 16, 2020 to May 19, 2020).

^b)^ p for ANOVA or *t*-test

^c)^ p for Wilcoxon rank sum test or Kruskal-Wallis test

Table 2 summarizes the fit indices of each model based on path analysis. Judging from CFI and RMSEA, model B best explained the relationships among the demographic, socioeconomic, and geographic variables, changes in work situation, perception of anxiety, and changes in walking and sedentary behaviors (CFI = 0.996, RMSEA = 0.057).

**Table 2:** Fit indices of each model.

**Model**	**CFI**^a)^	**RMSEA**^b)^
A	0.064	0.753
B	0.996	0.057
C	0.913	0.364
D	0.995	0.124

^a)^ Comparative Fit Index

^b)^ Root Mean Square Error of Approximation

**Fig. 4:**
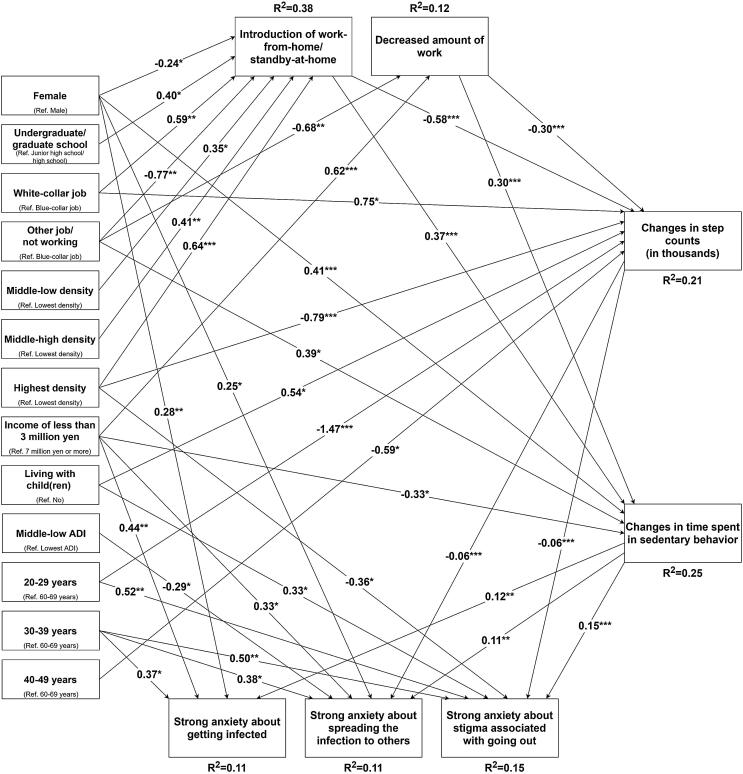
Coefficients of model B estimated by path analysis. Only significant paths and their coefficients among demographic, socioeconomic, and geographic variables, changes in work situation and perception of anxiety, and changes in walking and sedentary behaviors are shown.*** statistical significance at 0.1%; ** statistical significance at 1%; * statistical significance at 5%. CFI = 0.996, RMSEA = 0.057. Changes in step counts were determined by calculating the difference in the mean step counts between the pre-state of emergency (SoE) period (from February 19, 2020 to March 23, 2020) and the post-SoE period (from April 16, 2020 to May 19, 2020). The ordered categories of change in time spent in sedentary behavior were defined as follows: 1: significant reduction, 2: slight reduction, 3: no change, 4: slight rise, 5: significant rise.

Fig. 4 shows the significant paths in model B. Details of all the estimated coefficients of the models are provided in the appendix. Considering individual attributes and the changes in physical activity, respondents aged 20–29 years, aged 40–49 years, and living in the highest-density neighborhoods were more likely to experience reduced step counts. However, white-collar workers and living with child(ren) under 18 years were positively associated with differences in step counts and such individuals were less likely to experience reduced walking behavior. Moreover, females and workers in jobs other than white, gray, and blue-collar jobs or non-working participants were more likely to show increased sedentary behavior, while participants with household income less than 3 million yen were less likely to show increased sedentary behavior.

The work-from-home/standby-at-home group was positively associated with undergraduate/graduate school, and white-collar jobs, along with middle-low density, middle-high density, and highest density neighborhoods, while it was negatively associated with female respondents and the other jobs/not working group. Decreased amount of work was positively associated with an income of<3-million-yen group and negatively associated with the other jobs/not working group. The work-from-home/standby-at-home group and the decreased work group were associated with decreased step counts and increased time spent in sedentary behavior.

Female participants, age 30–39 years, and incomes<3 million yen were positively associated with strong anxiety about getting infected and about spreading the infection to others. Further, participants aged 20–29 years, aged 30–39 years, living with child(ren) under 18 years were positively associated with strong anxiety about the stigma of going out. Living in middle-low ADI were negatively associated with strong anxiety about spreading the infection to others and living in the highest density neighborhoods were negatively associated with the stigma of going out. Decreased step counts were associated with strong anxiety about spreading the infection to others or about the stigma of going out while increased time spent in sedentary behavior was associated with all anxiety variables.

## 4 Discussion

Several studies have documented decreased physical activity during the COVID-19 outbreak in Japan (Adachi et al., 2021, Hino and Asami, 2021, Makizako et al., 2021, Yamada et al., 2020). However, to the best of our knowledge, this is the first study to examine the association of changes in physical activity during the COVID-19 outbreak in Japan with the demographic, socioeconomic, and geographical attributes, and changes in work situation and perception of anxiety, simultaneously.

We found that overall, people became inactive during the first wave of the outbreak; based on the direct relationships estimated by path analysis, especially younger individuals and those living in high-density neighborhoods were more likely have decreased walking behavior. Previous studies have shown a similar trend in outing and walking behaviors (Adachi et al., 2021, Hanibuchi et al., 2021, Hino and Asami, 2021), and this could be attributed to more walks for daily activities before the pandemic among younger individuals and residents in urban area, thereby resulting in a significant decrease in the number of steps. Additionally, sedentary behavior clearly increased among females, similar to previous reports that showed that females became inactive during the outbreak in Japan (Hanibuchi et al., 2021, Hino and Asami, 2021, Makizako et al., 2021). Containment measures such as school closure or self-isolation may have increased the burden of housework on females (Hanibuchi et al., 2021, Power, 2020), making them more inactive.

As for the indirect relationships between individual attributes and the changes in physical activity via changes in work situation, individuals of higher SES, such as undergraduate/graduate school graduates and white-collar job workers, were more likely to implement preventive measures like work-from-home or standby-at-home, which were associated with decreased step counts and increased sedentary behavior. This could be because of decreased walking needed for commuting. Moreover, those living in high-density neighborhoods were more likely to implement work-from-home or standby-at-home strategies. This is expected as companies not requiring on-site work, such as information technology companies, are normally located in urban areas. Moreover, it is suggested that those with incomes<3 million yen were more likely to experience decreased work causing decreased step counts and increased sedentary behavior. Therefore, individuals with lower SES were more likely to experience economic problems and decreased physical activity simultaneously. Previous studies have revealed that unemployment is often associated with deterioration of mental health (Paul and Moser, 2009) and increased smoking and drinking behaviors (Montgomery et al., 1998). Individuals with lower SES with decreased work would have particularly high health risks during the pandemic.

Our results showed that changes in physical activity were associated with perception of anxiety, similar to several previous studies (Pieh et al., 2020, Silva et al., 2020). The model assuming that decreased physical activity causes increased anxiety better explained the comprehensive relationships compared to the model assuming that anxiety causes physical inactivity or the models assuming direct relationships between the work situation changes and anxiety. Increased anxiety during the pandemic is a crucial concern as a previous study demonstrated that COVID-19 related anxiety was strongly associated with functional impairment, alcohol or drug coping, negative religious coping, extreme hopelessness, and passive suicidal ideation (Lee, 2020). Our path model showed that females, younger individuals, and those living in high-density neighborhoods were more likely to experience decreased physical activity associated with anxiety. During the pandemic in Japan, several studies reported an increase in the number of females committing suicide (Nomura et al., 2021, Tanaka and Okamoto, 2021) and high urbanization to be associated with severe psychological distress and new-onset suicidal ideation (Okubo et al., 2021). These indicated that decreased physical activity could be one of the factors affecting such serious mental health problems during the pandemic in Japan.

Furthermore, the variables of anxiety regarding spreading the infection to others and stigma associated with going out were related to decreased walking behavior while the variable of anxiety about getting infected was not. This may be because in Japanese society with tight social norms (Gelfand et al., 2021), people are more anxious about disrupting social harmony by spreading the infection or going out than about getting infected. Although Gelfand et al. (2021) suggested that tightening social norms may mitigate the COVID-19 outbreak, in strict societies, social isolation due to decrease in opportunities to go out would increase people’s concerns about disturbing others or being criticized by others which may make people more inactive. Appropriate social norms should be considered based on overall health risks including infection, physical inactivity, and mental health.

This study has several limitations. First, the method of sample selection was not random, and the questionnaire has not been validated using external data; therefore, our findings are limited in their generalization. Second, a bias may have occurred due to the method of observation of walking behavior. Although we observed only the steps walked while the participants carried their iPhones, its frequency may vary according to personal attributes such as gender and age. We employed a simple method for quantitatively counting steps before the survey during the emergency; however, measurements by wearing an accelerometer at all times would have ideally reflected the changes in physical activity more accurately. Third, we attempted to explore the relationships between the changes in physical activity and individual attributes, the work situation changes, and perception of anxiety during the COVID-19 pandemic by comparing the path models based on several hypotheses; however, this cross-sectional study could not conclude the causality.

## 5 Conclusion

Physical inactivity during the COVID-19 pandemic is a serious concern causing various health problems. By examining the relationship of the changes in step count and time spent in sedentary behavior during the first wave of the outbreak in Japan to individual circumstances, the present study revealed that younger individuals, those living in high-density neighborhoods, and females were clearly associated with decreased walking behavior or increased sedentary behavior, and the changes in physical activity were associated with strong anxiety related to the pandemic. Further, while individuals with high SES were more likely to implement preventions such as work-from-home or standby-at-home, lower SES leads to decreased amounts of work, and both of those changes in work situation were related to decreased walking behavior and increased sedentary behavior. The health of people with low SES facing economic burden and females, younger individuals, and those living in urban areas who experience decreased physical activity should be continuously observed. Finally, considering that the pandemic is a changing, evolving situation, further analyses of changes in physical activity are warranted.

### CRediT authorship contribution statement

**Shohei Nagata:** Conceptualization, Formal analysis, Methodology, Software, Writing – original draft. **Hiroki M. Adachi:** Methodology, Software, Writing – review & editing. **Tomoya Hanibuchi:** Conceptualization, Data curation, Writing – review & editing, Funding acquisition. **Shiho Amagasa:** Conceptualization, Writing – review & editing. **Shigeru Inoue:** Conceptualization, Writing – review & editing, Funding acquisition. **Tomoki Nakaya:** Conceptualization, Writing – review & editing, Funding acquisition, Supervision.

## Declaration of Competing Interest

The authors declare that they have no known competing financial interests or personal relationships that could have appeared to influence the work reported in this paper.
