# Supplementary material for: Relationships among changes in walking and sedentary behaviors, individual attributes, changes in work situation, and anxiety during the COVID-19 pandemic in Japan
Source: Prev Med Rep. 2021 Nov 15;24:101640. doi: 10.1016/j.pmedr.2021.101640 (PMC8684034; doi:10.1016/j.pmedr.2021.101640)
Supplement: Supplementary data 1 [file mmc1.docx]

**Appendix**

Table A.1

Estimated coefficients of all paths to introduction of work-from-home/standby-at-home measures for model A

| From | Coef. | 95% CI | p |
| --- | --- | --- | --- |
| Gender (Ref. Male) |  |  |  |
| Female | -0.24 | -0.45 to -0.02 | 0.029* |
|  |  |  |  |
| Age (Ref. 60-69 years) |  |  |  |
| 20-29 years | 0.05 | -0.30 to 0.40 | 0.795 |
| 30-39 years | 0.15 | -0.18 to 0.49 | 0.363 |
| 40-49 years | 0.28 | -0.05 to 0.61 | 0.093 |
| 50-59 years | -0.04 | -0.37 to 0.29 | 0.793 |
|  |  |  |  |
| Chronic disease (Ref. No) |  |  |  |
| Yes | -0.02 | -0.28 to 0.23 | 0.856 |
|  |  |  |  |
| Educational status (Ref. Junior high school/high school) |  |  |  |
| Junior (technical) college/vocational school | 0.28 | -0.06 to 0.62 | 0.110 |
| Undergraduate/graduate school | 0.40 | 0.10 to 0.70 | 0.010* |
|  |  |  |  |
| Occupation (Ref. Blue-collar job) |  |  |  |
| White-collar job | 0.59 | 0.20 to 0.97 | 0.003** |
| Gray-collar job | 0.24 | -0.17 to 0.66 | 0.256 |
| Other/not working | -0.77 | -1.24 to -0.30 | <0.001*** |
|  |  |  |  |
| Household annual income (Ref. 7 million yen or more) |  |  |  |
| Less than 3 million yen | -0.13 | -0.48 to 0.22 | 0.458 |
| 3-7 million yen | -0.08 | -0.32 to 0.15 | 0.489 |
| Unknown | -0.09 | -0.43 to 0.24 | 0.586 |
|  |  |  |  |
| Living alone (Ref. No) |  |  |  |
| Yes | 0.16 | -0.12 to 0.45 | 0.257 |
|  |  |  |  |
| Living with child(ren) under 18 years (Ref. No) |  |  |  |
| Yes | -0.15 | -0.41 to 0.11 | 0.268 |
|  |  |  |  |
| Living with person(s) aged 65 years and older (Ref. No) |  |  |  |
| Yes | -0.10 | -0.40 to 0.19 | 0.490 |
|  |  |  |  |
| Neighborhood density (Ref. Lowest density) |  |  |  |
| Middle-low density | 0.35 | 0.07 to 0.64 | 0.016* |
| Middle-high density | 0.41 | 0.13 to 0.69 | 0.004** |
| Highest density | 0.64 | 0.34 to 0.94 | <0.001*** |
|  |  |  |  |
| Areal deprivation index (ADI) (Ref. Lowest ADI) |  |  |  |
| Middle-low ADI | 0.02 | -0.26 to 0.29 | 0.901 |
| Middle-high ADI | -0.12 | -0.40 to 0.17 | 0.429 |
| Highest ADI | -0.11 | -0.41 to 0.18 | 0.463 |

* Coef.: Coefficient and CI: confidence interval. “***,” “**,” and “*,” denote the statistical significance at 0.1%, 1%, and 5% levels, respectively. The sample size was 896.

Table A.2

Estimated coefficients of all paths to decreased amount of work for model A

| From | Coef. | 95% CI | p |
| --- | --- | --- | --- |
| Gender (Ref. Male) |  |  |  |
| Female | 0.09 | -0.13 to 0.31 | 0.437 |
|  |  |  |  |
| Age (Ref. 60-69 years) |  |  |  |
| 20-29 years | 0.07 | -0.30 to 0.43 | 0.723 |
| 30-39 years | 0.09 | -0.26 to 0.45 | 0.606 |
| 40-49 years | 0.08 | -0.27 to 0.43 | 0.653 |
| 50-59 years | 0.05 | -0.29 to 0.40 | 0.758 |
|  |  |  |  |
| Chronic disease (Ref. No) |  |  |  |
| Yes | 0.00 | -0.26 to 0.27 | 0.981 |
|  |  |  |  |
| Educational status (Ref. Junior high school/high school) |  |  |  |
| Junior (technical) college/vocational school | -0.01 | -0.34 to 0.31 | 0.938 |
| Undergraduate/graduate school | 0.05 | -0.24 to 0.33 | 0.757 |
|  |  |  |  |
| Occupation (Ref. Blue-collar job) |  |  |  |
| White-collar job | -0.18 | -0.56 to 0.19 | 0.336 |
| Gray-collar job | 0.11 | -0.29 to 0.50 | 0.599 |
| Other/not working | -0.68 | -1.09 to -0.27 | 0.001** |
|  |  |  |  |
| Household annual income (Ref. 7 million yen or more) |  |  |  |
| Less than 3 million yen | 0.62 | 0.27 to 0.96 | <0.001*** |
| 3-7 million yen | 0.22 | -0.03 to 0.47 | 0.084 |
| Unknown | 0.08 | -0.31 to 0.48 | 0.685 |
|  |  |  |  |
| Living alone (Ref. No) |  |  |  |
| Yes | 0.11 | -0.18 to 0.40 | 0.449 |
|  |  |  |  |
| Living with child(ren) under 18 years (Ref. No) |  |  |  |
| Yes | 0.03 | -0.24 to 0.31 | 0.813 |
|  |  |  |  |
| Living with person(s) aged 65 years and older (Ref. No) |  |  |  |
| Yes | 0.01 | -0.31 to 0.34 | 0.932 |
|  |  |  |  |
| Neighborhood density (Ref. Lowest density) |  |  |  |
| Middle-low density | -0.17 | -0.47 to 0.13 | 0.274 |
| Middle-high density | -0.03 | -0.32 to 0.27 | 0.859 |
| Highest density | 0.19 | -0.13 to 0.51 | 0.242 |
|  |  |  |  |
| Areal deprivation index (ADI) (Ref. Lowest ADI) |  |  |  |
| Middle-low ADI | 0.29 | -0.02 to 0.59 | 0.066 |
| Middle-high ADI | 0.25 | -0.07 to 0.56 | 0.123 |
| Highest ADI | 0.25 | -0.09 to 0.58 | 0.146 |

* Coef.: Coefficient and CI: confidence interval. “***,” “**,” and “*,” denote the statistical significance at 0.1%, 1%, and 5% levels, respectively. The sample size was 896.

Table A.3

Estimated coefficients of all paths to strong anxiety about getting infected for model A

| From | Coef. | 95% CI | p |
| --- | --- | --- | --- |
| Gender (Ref. Male) |  |  |  |
| Female | 0.32 | 0.12 to 0.52 | 0.002** |
|  |  |  |  |
| Age (Ref. 60-69 years) |  |  |  |
| 20-29 years | 0.03 | -0.30 to 0.35 | 0.873 |
| 30-39 years | 0.40 | 0.10 to 0.70 | 0.009** |
| 40-49 years | 0.28 | -0.02 to 0.57 | 0.067 |
| 50-59 years | 0.28 | -0.01 to 0.57 | 0.057 |
|  |  |  |  |
| Chronic disease (Ref. No) |  |  |  |
| Yes | 0.20 | -0.01 to 0.41 | 0.065 |
|  |  |  |  |
| Educational status (Ref. Junior high school/high school) |  |  |  |
| Junior (technical) college/vocational school | 0.13 | -0.15 to 0.41 | 0.354 |
| Undergraduate/graduate school | 0.11 | -0.14 to 0.36 | 0.399 |
|  |  |  |  |
| Occupation (Ref. Blue-collar job) |  |  |  |
| White-collar job | -0.02 | -0.38 to 0.33 | 0.899 |
| Gray-collar job | 0.09 | -0.29 to 0.48 | 0.638 |
| Other/not working | 0.06 | -0.33 to 0.44 | 0.772 |
|  |  |  |  |
| Household annual income (Ref. 7 million yen or more) |  |  |  |
| Less than 3 million yen | 0.42 | 0.10 to 0.73 | 0.010* |
| 3-7 million yen | 0.09 | -0.13 to 0.31 | 0.420 |
| Unknown | 0.06 | -0.27 to 0.38 | 0.726 |
|  |  |  |  |
| Living alone (Ref. No) |  |  |  |
| Yes | -0.19 | -0.46 to 0.09 | 0.190 |
|  |  |  |  |
| Living with child(ren) under 18 years (Ref. No) |  |  |  |
| Yes | 0.16 | -0.08 to 0.40 | 0.187 |
|  |  |  |  |
| Living with person(s) aged 65 years and older (Ref. No) |  |  |  |
| Yes | -0.13 | -0.41 to 0.14 | 0.347 |
|  |  |  |  |
| Neighborhood density (Ref. Lowest density) |  |  |  |
| Middle-low density | 0.20 | -0.05 to 0.46 | 0.112 |
| Middle-high density | 0.00 | -0.26 to 0.26 | 0.990 |
| Highest density | -0.02 | -0.29 to 0.25 | 0.876 |
|  |  |  |  |
| Areal deprivation index (ADI) (Ref. Lowest ADI) |  |  |  |
| Middle-low ADI | -0.08 | -0.33 to 0.18 | 0.551 |
| Middle-high ADI | -0.01 | -0.28 to 0.25 | 0.919 |
| Highest ADI | -0.01 | -0.27 to 0.26 | 0.956 |

* Coef.: Coefficient and CI: confidence interval. “***,” “**,” and “*,” denote the statistical significance at 0.1%, 1%, and 5% levels, respectively. The sample size was 896.

Table A.4

Estimated coefficients of all paths to strong anxiety about spreading the infection to others for model A

| From | Coef. | 95% CI | p |
| --- | --- | --- | --- |
| Gender (Ref. Male) |  |  |  |
| Female | 0.30 | 0.09 to 0.50 | 0.005** |
|  |  |  |  |
| Age (Ref. 60-69 years) |  |  |  |
| 20-29 years | 0.13 | -0.20 to 0.46 | 0.445 |
| 30-39 years | 0.42 | 0.10 to 0.74 | 0.009** |
| 40-49 years | 0.20 | -0.12 to 0.51 | 0.227 |
| 50-59 years | 0.15 | -0.17 to 0.47 | 0.366 |
|  |  |  |  |
| Chronic disease (Ref. No) |  |  |  |
| Yes | 0.18 | -0.04 to 0.40 | 0.114 |
|  |  |  |  |
| Educational status (Ref. Junior high school/high school) |  |  |  |
| Junior (technical) college/vocational school | 0.13 | -0.16 to 0.42 | 0.377 |
| Undergraduate/graduate school | 0.15 | -0.12 to 0.42 | 0.274 |
|  |  |  |  |
| Occupation (Ref. Blue-collar job) |  |  |  |
| White-collar job | -0.19 | -0.56 to 0.18 | 0.320 |
| Gray-collar job | 0.01 | -0.39 to 0.41 | 0.970 |
| Other/not working | -0.04 | -0.44 to 0.36 | 0.843 |
|  |  |  |  |
| Household annual income (Ref. 7 million yen or more) |  |  |  |
| Less than 3 million yen | 0.30 | -0.02 to 0.63 | 0.065 |
| 3-7 million yen | 0.04 | -0.19 to 0.28 | 0.722 |
| Unknown | -0.05 | -0.39 to 0.29 | 0.779 |
|  |  |  |  |
| Living alone (Ref. No) |  |  |  |
| Yes | -0.02 | -0.30 to 0.27 | 0.915 |
|  |  |  |  |
| Living with child(ren) under 18 years (Ref. No) |  |  |  |
| Yes | 0.07 | -0.18 to 0.31 | 0.603 |
|  |  |  |  |
| Living with person(s) aged 65 years and older (Ref. No) |  |  |  |
| Yes | -0.16 | -0.46 to 0.15 | 0.308 |
|  |  |  |  |
| Neighborhood density (Ref. Lowest density) |  |  |  |
| Middle-low density | 0.18 | -0.08 to 0.45 | 0.175 |
| Middle-high density | 0.04 | -0.24 to 0.31 | 0.803 |
| Highest density | -0.04 | -0.34 to 0.25 | 0.766 |
|  |  |  |  |
| Areal deprivation index (ADI) (Ref. Lowest ADI) |  |  |  |
| Middle-low ADI | -0.26 | -0.53 to 0.01 | 0.057 |
| Middle-high ADI | -0.01 | -0.29 to 0.26 | 0.920 |
| Highest ADI | -0.07 | -0.34 to 0.21 | 0.618 |

* Coef.: Coefficient and CI: confidence interval. “***,” “**,” and “*,” denote the statistical significance at 0.1%, 1%, and 5% levels, respectively. The sample size was 896.

Table A.5

Estimated coefficients of all paths to strong anxiety about stigma associated with going out for model A

| From | Coef. | 95% CI | p |
| --- | --- | --- | --- |
| Gender (Ref. Male) |  |  |  |
| Female | 0.02 | -0.23 to 0.27 | 0.896 |
|  |  |  |  |
| Age (Ref. 60-69 years) |  |  |  |
| 20-29 years | 0.66 | 0.28 to 1.04 | <0.001*** |
| 30-39 years | 0.54 | 0.17 to 0.92 | 0.004** |
| 40-49 years | 0.28 | -0.11 to 0.67 | 0.154 |
| 50-59 years | 0.35 | -0.03 to 0.73 | 0.07 |
|  |  |  |  |
| Chronic disease (Ref. No) |  |  |  |
| Yes | 0.17 | -0.09 to 0.42 | 0.198 |
|  |  |  |  |
| Educational status (Ref. Junior high school/high school) |  |  |  |
| Junior (technical) college/vocational school | 0.29 | -0.04 to 0.62 | 0.080 |
| Undergraduate/graduate school | 0.03 | -0.28 to 0.34 | 0.872 |
|  |  |  |  |
| Occupation (Ref. Blue-collar job) |  |  |  |
| White-collar job | -0.13 | -0.53 to 0.26 | 0.511 |
| Gray-collar job | -0.14 | -0.56 to 0.28 | 0.517 |
| Other/not working | -0.04 | -0.47 to 0.38 | 0.839 |
|  |  |  |  |
| Household annual income (Ref. 7 million yen or more) |  |  |  |
| Less than 3 million yen | 0.11 | -0.26 to 0.47 | 0.560 |
| 3-7 million yen | -0.19 | -0.45 to 0.08 | 0.166 |
| Unknown | -0.12 | -0.50 to 0.26 | 0.534 |
|  |  |  |  |
| Living alone (Ref. No) |  |  |  |
| Yes | 0.17 | -0.16 to 0.49 | 0.312 |
|  |  |  |  |
| Living with child(ren) under 18 years (Ref. No) |  |  |  |
| Yes | 0.27 | -0.01 to 0.55 | 0.055 |
|  |  |  |  |
| Living with person(s) aged 65 years and older (Ref. No) |  |  |  |
| Yes | -0.11 | -0.45 to 0.22 | 0.506 |
|  |  |  |  |
| Neighborhood density (Ref. Lowest density) |  |  |  |
| Middle-low density | 0.08 | -0.21 to 0.38 | 0.589 |
| Middle-high density | -0.08 | -0.39 to 0.23 | 0.597 |
| Highest density | -0.23 | -0.56 to 0.10 | 0.172 |
|  |  |  |  |
| Areal deprivation index (ADI) (Ref. Lowest ADI) |  |  |  |
| Middle-low ADI | 0.03 | -0.28 to 0.35 | 0.840 |
| Middle-high ADI | 0.21 | -0.10 to 0.52 | 0.190 |
| Highest ADI | 0.15 | -0.16 to 0.47 | 0.340 |

* Coef.: Coefficient and CI: confidence interval. “***,” “**,” and “*,” denote the statistical significance at 0.1%, 1%, and 5% levels, respectively. The sample size was 896.

Table A.6

Estimated coefficients of all paths to the changes in step counts (in thousands) between the pre-SoE and post-SoE periods for model A

| From | Coef. | 95% CI | p |
| --- | --- | --- | --- |
| Gender (Ref. Male) |  |  |  |
| Female | 0.04 | -0.37 to 0.44 | 0.863 |
|  |  |  |  |
| Age (Ref. 60-69 years) |  |  |  |
| 20-29 years | -1.23 | -1.80 to -0.65 | <0.001*** |
| 30-39 years | -0.03 | -0.69 to 0.62 | 0.923 |
| 40-49 years | -0.44 | -1.04 to 0.15 | 0.144 |
| 50-59 years | -0.15 | -0.73 to 0.44 | 0.628 |
|  |  |  |  |
| Chronic disease (Ref. No) |  |  |  |
| Yes | 0.08 | -0.36 to 0.52 | 0.722 |
|  |  |  |  |
| Educational status (Ref. Junior high school/high school) |  |  |  |
| Junior (technical) college/vocational school | 0.24 | -0.39 to 0.86 | 0.457 |
| Undergraduate/graduate school | -0.13 | -0.69 to 0.43 | 0.648 |
|  |  |  |  |
| Occupation (Ref. Blue-collar job) |  |  |  |
| White-collar job | 0.61 | -0.01 to 1.23 | 0.054 |
| Gray-collar job | 0.31 | -0.33 to 0.96 | 0.339 |
| Other/not working | -0.10 | -0.77 to 0.58 | 0.776 |
|  |  |  |  |
| Household annual income (Ref. 7 million yen or more) |  |  |  |
| Less than 3 million yen | 0.27 | -0.31 to 0.86 | 0.360 |
| 3-7 million yen | -0.03 | -0.49 to 0.42 | 0.885 |
| Unknown | -0.17 | -0.77 to 0.42 | 0.571 |
|  |  |  |  |
| Living alone (Ref. No) |  |  |  |
| Yes | 0.40 | -0.10 to 0.91 | 0.119 |
|  |  |  |  |
| Living with child(ren) under 18 years (Ref. No) |  |  |  |
| Yes | 0.69 | 0.18 to 1.19 | 0.008** |
|  |  |  |  |
| Living with person(s) aged 65 years and older (Ref. No) |  |  |  |
| Yes | 0.00 | -0.56 to 0.57 | 0.988 |
|  |  |  |  |
| Neighborhood density (Ref. Lowest density) |  |  |  |
| Middle-low density | 0.14 | -0.36 to 0.64 | 0.596 |
| Middle-high density | -0.41 | -0.89 to 0.07 | 0.098 |
| Highest density | -0.99 | -1.47 to -0.50 | <0.001*** |
|  |  |  |  |
| Areal deprivation index (ADI) (Ref. Lowest ADI) |  |  |  |
| Middle-low ADI | -0.35 | -0.81 to 0.12 | 0.146 |
| Middle-high ADI | 0.14 | -0.36 to 0.64 | 0.575 |
| Highest ADI | 0.19 | -0.33 to 0.71 | 0.473 |

Table A.6 (continued)

| From | Coef. | 95% CI | p |
| --- | --- | --- | --- |
| Introduction of work-from-home/standby-at-home (Ref. No) |  |  |  |
| Yes | -0.46 | -0.59 to -0.33 | <0.001*** |
|  |  |  |  |
| Decreased amount of work (Ref. No) |  |  |  |
| Yes | -0.14 | -0.30 to 0.03 | 0.104 |
|  |  |  |  |
| Strong anxiety about getting infected (Ref. No) |  |  |  |
| Yes | -0.16 | -0.28 to -0.03 | 0.014* |
|  |  |  |  |
| Strong anxiety about spreading the infection to others (Ref. No) |  |  |  |
| Yes | -0.30 | -0.40 to -0.21 | <0.001*** |
|  |  |  |  |
| Strong anxiety about stigma associated with going out (Ref. No) |  |  |  |
| Yes | -0.33 | -0.42 to -0.24 | <0.001*** |

* Coef.: Coefficient and CI: confidence interval. “***,” “**,” and “*,” denote the statistical significance at 0.1%, 1%, and 5% levels, respectively. The sample size was 896.

Table A.7

Estimated coefficients of all paths to the changes in time spent in sedentary behavior for model A

| From | Coef. | 95% CI | p |
| --- | --- | --- | --- |
| Gender (Ref. Male) |  |  |  |
| Female | 0.30 | 0.11 to 0.49 | 0.002** |
|  |  |  |  |
| Age (Ref. 60-69 years) |  |  |  |
| 20-29 years | 0.10 | -0.20 to 0.39 | 0.530 |
| 30-39 years | -0.22 | -0.53 to 0.08 | 0.157 |
| 40-49 years | -0.12 | -0.42 to 0.17 | 0.418 |
| 50-59 years | -0.21 | -0.50 to 0.09 | 0.165 |
|  |  |  |  |
| Chronic disease (Ref. No) |  |  |  |
| Yes | 0.00 | -0.21 to 0.20 | 0.990 |
|  |  |  |  |
| Educational status (Ref. Junior high school/high school) |  |  |  |
| Junior (technical) college/vocational school | -0.12 | -0.39 to 0.14 | 0.366 |
| Undergraduate/graduate school | 0.08 | -0.17 to 0.32 | 0.544 |
|  |  |  |  |
| Occupation (Ref. Blue-collar job) |  |  |  |
| White-collar job | -0.18 | -0.52 to 0.15 | 0.285 |
| Gray-collar job | -0.15 | -0.51 to 0.21 | 0.415 |
| Other/not working | 0.30 | -0.06 to 0.67 | 0.105 |
|  |  |  |  |
| Household annual income (Ref. 7 million yen or more) |  |  |  |
| Less than 3 million yen | -0.44 | -0.73 to -0.14 | 0.004** |
| 3-7 million yen | 0.07 | -0.14 to 0.28 | 0.495 |
| Unknown | -0.22 | -0.52 to 0.08 | 0.157 |
|  |  |  |  |
| Living alone (Ref. No) |  |  |  |
| Yes | -0.14 | -0.39 to 0.10 | 0.253 |
|  |  |  |  |
| Living with child(ren) under 18 years (Ref. No) |  |  |  |
| Yes | -0.19 | -0.42 to 0.04 | 0.109 |
|  |  |  |  |
| Living with person(s) aged 65 years and older (Ref. No) |  |  |  |
| Yes | -0.01 | -0.26 to 0.23 | 0.930 |
|  |  |  |  |
| Neighborhood density (Ref. Lowest density) |  |  |  |
| Middle-low density | 0.02 | -0.22 to 0.26 | 0.872 |
| Middle-high density | 0.03 | -0.23 to 0.29 | 0.810 |
| Highest density | 0.18 | -0.07 to 0.44 | 0.156 |
|  |  |  |  |
| Areal deprivation index (ADI) (Ref. Lowest ADI) |  |  |  |
| Middle-low ADI | 0.11 | -0.14 to 0.35 | 0.399 |
| Middle-high ADI | -0.16 | -0.41 to 0.08 | 0.198 |
| Highest ADI | -0.12 | -0.37 to 0.14 | 0.365 |

Table A.7 (continued)

| From | Coef. | 95% CI | p |
| --- | --- | --- | --- |
| Introduction of work-from-home/standby-at-home (Ref. No) |  |  |  |
| Yes | 0.30 | 0.22 to 0.38 | <0.001*** |
|  |  |  |  |
| Decreased amount of work (Ref. No) |  |  |  |
| Yes | 0.25 | 0.15 to 0.34 | <0.001*** |
|  |  |  |  |
| Strong anxiety about getting infected (Ref. No) |  |  |  |
| Yes | 0.15 | 0.06 to 0.24 | <0.001*** |
|  |  |  |  |
| Strong anxiety about spreading the infection to others (Ref. No) |  |  |  |
| Yes | 0.15 | 0.06 to 0.24 | <0.001*** |
|  |  |  |  |
| Strong anxiety about stigma associated with going out (Ref. No) |  |  |  |
| Yes | 0.21 | 0.12 to 0.31 | <0.001*** |

* Coef.: Coefficient and CI: confidence interval. “***,” “**,” and “*,” denote the statistical significance at 0.1%, 1%, and 5% levels, respectively. The sample size was 896. The ordered categories of change in time spent in sedentary behavior were defined as follows: 1: significant reduction, 2: slight reduction, 3: no change, 4: slight rise, 5: significant rise.

Table A.8

Estimated coefficients of all paths to introduction of work-from-home/standby-at-home measures for model B

| From | Coef. | 95% CI | p |
| --- | --- | --- | --- |
| Gender (Ref. Male) |  |  |  |
| Female | -0.24 | -0.45 to -0.02 | 0.029* |
|  |  |  |  |
| Age (Ref. 60-69 years) |  |  |  |
| 20-29 years | 0.05 | -0.30 to 0.40 | 0.796 |
| 30-39 years | 0.15 | -0.18 to 0.49 | 0.363 |
| 40-49 years | 0.28 | -0.05 to 0.61 | 0.093 |
| 50-59 years | -0.04 | -0.37 to 0.29 | 0.793 |
|  |  |  |  |
| Chronic disease (Ref. No) |  |  |  |
| Yes | -0.02 | -0.28 to 0.23 | 0.856 |
|  |  |  |  |
| Educational attainment (Ref. Junior high school/high school) |  |  |  |
| Junior (technical) college/vocational school | 0.28 | -0.06 to 0.62 | 0.11 |
| Undergraduate/graduate school | 0.40 | 0.10 to 0.70 | 0.01* |
|  |  |  |  |
| Occupation (Ref. Blue-collar job) |  |  |  |
| White-collar job | 0.59 | 0.20 to 0.97 | 0.003** |
| Gray-collar job | 0.24 | -0.17 to 0.66 | 0.256 |
| Other/not working | -0.77 | -1.24 to -0.30 | 0.001** |
|  |  |  |  |
| Household annual income (Ref. 7 million yen or more) |  |  |  |
| Less than 3 million yen | -0.13 | -0.48 to 0.22 | 0.458 |
| 3-7 million yen | -0.08 | -0.32 to 0.15 | 0.489 |
| Unknown | -0.09 | -0.43 to 0.24 | 0.586 |
|  |  |  |  |
| Living alone (Ref. No) |  |  |  |
| Yes | 0.16 | -0.12 to 0.45 | 0.257 |
|  |  |  |  |
| Living with child(ren) under 18 years (Ref. No) |  |  |  |
| Yes | -0.15 | -0.41 to 0.11 | 0.268 |
|  |  |  |  |
| Living with person(s) aged 65 years and older (Ref. No) |  |  |  |
| Yes | -0.10 | -0.40 to 0.19 | 0.490 |
|  |  |  |  |
| Neighborhood density (Ref. Lowest density) |  |  |  |
| Middle-low density | 0.35 | 0.07 to 0.64 | 0.016* |
| Middle-high density | 0.41 | 0.13 to 0.69 | 0.004** |
| Highest density | 0.64 | 0.34 to 0.94 | <0.001*** |
|  |  |  |  |
| Areal deprivation index (ADI) (Ref. Lowest ADI) |  |  |  |
| Middle-low ADI | 0.02 | -0.26 to 0.29 | 0.901 |
| Middle-high ADI | -0.12 | -0.40 to 0.17 | 0.429 |
| Highest ADI | -0.11 | -0.41 to 0.18 | 0.463 |

* Coef.: Coefficient and CI: confidence interval. “***,” “**,” and “*,” denote the statistical significance at 0.1%, 1%, and 5% levels, respectively. The sample size was 896.

Table A.9

Estimated coefficients of all paths to decreased amount of work for model B

| From | Coef. | 95% CI | p |
| --- | --- | --- | --- |
| Gender (Ref. Male) |  |  |  |
| Female | 0.09 | -0.13 to 0.31 | 0.437 |
|  |  |  |  |
| Age (Ref. 60-69 years) |  |  |  |
| 20-29 years | 0.07 | -0.30 to 0.43 | 0.723 |
| 30-39 years | 0.09 | -0.26 to 0.45 | 0.606 |
| 40-49 years | 0.08 | -0.27 to 0.43 | 0.653 |
| 50-59 years | 0.05 | -0.29 to 0.40 | 0.758 |
|  |  |  |  |
| Chronic disease (Ref. No) |  |  |  |
| Yes | 0.00 | -0.26 to 0.27 | 0.981 |
|  |  |  |  |
| Educational attainment (Ref. Junior high school/high school) |  |  |  |
| Junior (technical) college/vocational school | -0.01 | -0.34 to 0.31 | 0.938 |
| Undergraduate/graduate school | 0.05 | -0.24 to 0.33 | 0.757 |
|  |  |  |  |
| Occupation (Ref. Blue-collar job) |  |  |  |
| White-collar job | -0.18 | -0.56 to 0.19 | 0.336 |
| Gray-collar job | 0.11 | -0.29 to 0.50 | 0.599 |
| Other/not working | -0.68 | -1.09 to -0.27 | 0.001** |
|  |  |  |  |
| Household annual income (Ref. 7 million yen or more) |  |  |  |
| Less than 3 million yen | 0.62 | 0.27 to 0.96 | <0.001*** |
| 3-7 million yen | 0.22 | -0.03 to 0.47 | 0.084 |
| Unknown | 0.08 | -0.31 to 0.48 | 0.685 |
|  |  |  |  |
| Living alone (Ref. No) |  |  |  |
| Yes | 0.11 | -0.18 to 0.40 | 0.449 |
|  |  |  |  |
| Living with child(ren) under 18 years (Ref. No) |  |  |  |
| Yes | 0.03 | -0.24 to 0.31 | 0.813 |
|  |  |  |  |
| Living with person(s) aged 65 years and older (Ref. No) |  |  |  |
| Yes | 0.01 | -0.31 to 0.34 | 0.932 |
|  |  |  |  |
| Neighborhood density (Ref. Lowest density) |  |  |  |
| Middle-low density | -0.17 | -0.47 to 0.13 | 0.274 |
| Middle-high density | -0.03 | -0.32 to 0.27 | 0.859 |
| Highest density | 0.19 | -0.13 to 0.51 | 0.242 |
|  |  |  |  |
| Areal deprivation index (ADI) (Ref. Lowest ADI) |  |  |  |
| Middle-low ADI | 0.29 | -0.02 to 0.59 | 0.066 |
| Middle-high ADI | 0.25 | -0.07 to 0.56 | 0.123 |
| Highest ADI | 0.25 | -0.09 to 0.58 | 0.146 |

* Coef.: Coefficient and CI: confidence interval. “***,” “**,” and “*,” denote the statistical significance at 0.1%, 1%, and 5% levels, respectively. The sample size was 896.

Table A.10

Estimated coefficients of all paths to the changes in step counts (in thousands) between the pre-SoE and post-SoE periods for model B

| From | Coef. | 95% CI | p |
| --- | --- | --- | --- |
| Gender (Ref. Male) |  |  |  |
| Female | -0.12 | -0.50 to 0.25 | 0.521 |
|  |  |  |  |
| Age (Ref. 60-69 years) |  |  |  |
| 20-29 years | -1.47 | -2.00 to -0.94 | <0.001*** |
| 30-39 years | -0.37 | -0.99 to 0.25 | 0.246 |
| 40-49 years | -0.59 | -1.17 to -0.02 | 0.043* |
| 50-59 years | -0.35 | -0.92 to 0.22 | 0.234 |
|  |  |  |  |
| Chronic disease (Ref. No) |  |  |  |
| Yes | -0.06 | -0.49 to 0.36 | 0.773 |
|  |  |  |  |
| Educational attainment (Ref. Junior high school/high school) |  |  |  |
| Junior (technical) college/vocational school | 0.11 | -0.49 to 0.71 | 0.721 |
| Undergraduate/graduate school | -0.15 | -0.69 to 0.39 | 0.589 |
|  |  |  |  |
| Occupation (Ref. Blue-collar job) |  |  |  |
| White-collar job | 0.75 | 0.17 to 1.33 | 0.012* |
| Gray-collar job | 0.39 | -0.20 to 0.98 | 0.196 |
| Other/not working | -0.28 | -0.93 to 0.37 | 0.397 |
|  |  |  |  |
| Household annual income (Ref. 7 million yen or more) |  |  |  |
| Less than 3 million yen | 0.17 | -0.38 to 0.71 | 0.551 |
| 3-7 million yen | 0.03 | -0.41 to 0.47 | 0.903 |
| Unknown | -0.12 | -0.70 to 0.45 | 0.673 |
|  |  |  |  |
| Living alone (Ref. No) |  |  |  |
| Yes | 0.42 | -0.06 to 0.89 | 0.086 |
|  |  |  |  |
| Living with child(ren) under 18 years (Ref. No) |  |  |  |
| Yes | 0.54 | 0.05 to 1.03 | 0.030* |
|  |  |  |  |
| Living with person(s) aged 65 years and older (Ref. No) |  |  |  |
| Yes | 0.10 | -0.45 to 0.65 | 0.718 |
|  |  |  |  |
| Neighborhood density (Ref. Lowest density) |  |  |  |
| Middle-low density | 0.03 | -0.44 to 0.51 | 0.889 |
| Middle-high density | -0.35 | -0.83 to 0.13 | 0.157 |
| Highest density | -0.79 | -1.25 to -0.32 | <0.001*** |
|  |  |  |  |
| Areal deprivation index (ADI) (Ref. Lowest ADI) |  |  |  |
| Middle-low ADI | -0.22 | -0.67 to 0.24 | 0.354 |
| Middle-high ADI | 0.11 | -0.38 to 0.60 | 0.664 |
| Highest ADI | 0.19 | -0.31 to 0.69 | 0.459 |

| Table A.10 (continued) |  |  |  |
| --- | --- | --- | --- |
| From | Coef. | 95% CI | p |
| Introduction of working-from-home/standby at home (Ref. No) |  |  |  |
| Yes | -0.58 | -0.71 to -0.45 | <0.001*** |
|  |  |  |  |
| Decreased amount of work (Ref. No) |  |  |  |
| Yes | -0.30 | -0.46 to -0.14 | <0.001*** |

* Coef.: Coefficient and CI: confidence interval. “***,” “**,” and “*,” denote the statistical significance at 0.1%, 1%, and 5% levels, respectively. The sample size was 896.

Table A.11

Estimated coefficients of all paths to the changes in time spent in sedentary behavior for model B

| From | Coef. | 95% CI | p |
| --- | --- | --- | --- |
| Gender (Ref. Male) |  |  |  |
| Female | 0.41 | 0.24 to 0.58 | <0.001*** |
|  |  |  |  |
| Age (Ref. 60-69 years) |  |  |  |
| 20-29 years | 0.25 | -0.03 to 0.53 | 0.075 |
| 30-39 years | 0.01 | -0.27 to 0.28 | 0.968 |
| 40-49 years | -0.01 | -0.30 to 0.27 | 0.920 |
| 50-59 years | -0.07 | -0.33 to 0.20 | 0.629 |
|  |  |  |  |
| Chronic disease (Ref. No) |  |  |  |
| Yes | 0.09 | -0.10 to 0.29 | 0.347 |
|  |  |  |  |
| Educational attainment (Ref. Junior high school/high school) |  |  |  |
| Junior (technical) college/vocational school | -0.04 | -0.29 to 0.21 | 0.760 |
| Undergraduate/graduate school | 0.09 | -0.15 to 0.33 | 0.462 |
|  |  |  |  |
| Occupation (Ref. Blue-collar job) |  |  |  |
| White-collar job | -0.28 | -0.60 to 0.05 | 0.099 |
| Gray-collar job | -0.19 | -0.52 to 0.15 | 0.282 |
| Other/not working | 0.39 | 0.03 to 0.75 | 0.033* |
|  |  |  |  |
| Household annual income (Ref. 7 million yen or more) |  |  |  |
| Less than 3 million yen | -0.33 | -0.60 to -0.06 | 0.019* |
| 3-7 million yen | 0.05 | -0.15 to 0.24 | 0.645 |
| Unknown | -0.24 | -0.53 to 0.05 | 0.105 |
|  |  |  |  |
| Living alone (Ref. No) |  |  |  |
| Yes | -0.16 | -0.39 to 0.07 | 0.183 |
|  |  |  |  |
| Living with child(ren) under 18 years (Ref. No) |  |  |  |
| Yes | -0.09 | -0.31 to 0.13 | 0.429 |
|  |  |  |  |
| Living with person(s) aged 65 years and older (Ref. No) |  |  |  |
| Yes | -0.07 | -0.30 to 0.15 | 0.521 |
|  |  |  |  |
| Neighborhood density (Ref. Lowest density) |  |  |  |
| Middle-low density | 0.08 | -0.16 to 0.32 | 0.503 |
| Middle-high density | 0.00 | -0.25 to 0.23 | 0.941 |
| Highest density | 0.07 | -0.17 to 0.31 | 0.579 |
|  |  |  |  |
| Areal deprivation index (ADI) (Ref. Lowest ADI) |  |  |  |
| Middle-low ADI | 0.04 | -0.19 to 0.27 | 0.714 |
| Middle-high ADI | -0.13 | -0.36 to 0.10 | 0.278 |
| Highest ADI | -0.10 | -0.34 to 0.13 | 0.389 |

Table A.11 (continued)

| From | Coef. | 95% CI | p |
| --- | --- | --- | --- |
| Introduction of working-from-home/standby at home (Ref. No) |  |  |  |
| Yes | 0.37 | 0.29 to 0.45 | <0.001*** |
|  |  |  |  |
| Decreased amount of work (Ref. No) |  |  |  |
| Yes | 0.30 | 0.21 to 0.40 | <0.001*** |

* Coef.: Coefficient and CI: confidence interval. “***,” “**,” and “*,” denote the statistical significance at 0.1%, 1%, and 5% levels, respectively. The sample size was 896. The ordered categories of change in time spent in sedentary behavior were defined as follows: 1: significant reduction, 2: slight reduction, 3: no change, 4: slight rise, 5: significant rise.

Table A.12

Estimated coefficients of all paths to strong anxiety about getting infected for model B

| From | Coef. | 95% CI | p |
| --- | --- | --- | --- |
| Gender (Ref. Male) |  |  |  |
| Female | 0.28 | 0.08 to 0.48 | 0.007** |
|  |  |  |  |
| Age (Ref. 60-69 years) |  |  |  |
| 20-29 years | -0.05 | -0.38 to 0.28 | 0.761 |
| 30-39 years | 0.37 | 0.08 to 0.67 | 0.013* |
| 40-49 years | 0.24 | -0.05 to 0.53 | 0.107 |
| 50-59 years | 0.28 | -0.01 to 0.57 | 0.057 |
|  |  |  |  |
| Chronic disease (Ref. No) |  |  |  |
| Yes | 0.19 | -0.02 to 0.40 | 0.080 |
|  |  |  |  |
| Educational attainment (Ref. Junior high school/high school) |  |  |  |
| Junior (technical) college/vocational school | 0.12 | -0.15 to 0.40 | 0.388 |
| Undergraduate/graduate school | 0.07 | -0.18 to 0.31 | 0.605 |
|  |  |  |  |
| Occupation (Ref. Blue-collar job) |  |  |  |
| White-collar job | 0.00 | -0.35 to 0.36 | 0.985 |
| Gray-collar job | 0.11 | -0.28 to 0.49 | 0.589 |
| Other/not working | 0.08 | -0.30 to 0.46 | 0.683 |
|  |  |  |  |
| Household annual income (Ref. 7 million yen or more) |  |  |  |
| Less than 3 million yen | 0.44 | 0.12 to 0.76 | 0.006** |
| 3-7 million yen | 0.08 | -0.14 to 0.30 | 0.471 |
| Unknown | 0.09 | -0.23 to 0.41 | 0.597 |
|  |  |  |  |
| Living alone (Ref. No) |  |  |  |
| Yes | -0.17 | -0.45 to 0.11 | 0.232 |
|  |  |  |  |
| Living with child(ren) under 18 years (Ref. No) |  |  |  |
| Yes | 0.19 | -0.04 to 0.43 | 0.108 |
|  |  |  |  |
| Living with person(s) aged 65 years and older (Ref. No) |  |  |  |
| Yes | -0.11 | -0.39 to 0.16 | 0.409 |
|  |  |  |  |
| Neighborhood density (Ref. Lowest density) |  |  |  |
| Middle-low density | 0.18 | -0.07 to 0.43 | 0.155 |
| Middle-high density | -0.03 | -0.29 to 0.22 | 0.793 |
| Highest density | -0.10 | -0.37 to 0.17 | 0.470 |
|  |  |  |  |
| Areal deprivation index (ADI) (Ref. Lowest ADI) |  |  |  |
| Middle-low ADI | -0.10 | -0.36 to 0.15 | 0.429 |
| Middle-high ADI | 0.00 | -0.26 to 0.26 | 0.994 |
| Highest ADI | 0.01 | -0.26 to 0.27 | 0.964 |

Table A.12 (continued)

| From | Coef. | 95% CI | p |
| --- | --- | --- | --- |
| Changes in step counts (in thousands) between the pre-SoE and the post-SoE periods | -0.03 | -0.05 to 0.00 | 0.052 |
|  |  |  |  |
| Changes in time spent sedentary behavior during the COVID-19 outbreak | 0.12 | 0.05 to 0.20 | 0.001** |

* Coef.: Coefficient and CI: confidence interval. “***,” “**,” and “*,” denote the statistical significance at 0.1%, 1%, and 5% levels, respectively. The sample size was 896.

Table A.13

Estimated coefficients of all paths to strong anxiety about spreading the infection to others for model B

| From | Coef. | 95% CI | p |
| --- | --- | --- | --- |
| Gender (Ref. Male) |  |  |  |
| Female | 0.25 | 0.05 to 0.46 | 0.017* |
|  |  |  |  |
| Age (Ref. 60-69 years) |  |  |  |
| 20-29 years | 0.01 | -0.33 to 0.34 | 0.965 |
| 30-39 years | 0.38 | 0.07 to 0.70 | 0.018* |
| 40-49 years | 0.14 | -0.18 to 0.45 | 0.394 |
| 50-59 years | 0.13 | -0.18 to 0.45 | 0.409 |
|  |  |  |  |
| Chronic disease (Ref. No) |  |  |  |
| Yes | 0.17 | -0.05 to 0.39 | 0.137 |
|  |  |  |  |
| Educational attainment (Ref. Junior high school/high school) |  |  |  |
| Junior (technical) college/vocational school | 0.12 | -0.17 to 0.41 | 0.411 |
| Undergraduate/graduate school | 0.10 | -0.17 to 0.36 | 0.47 |
|  |  |  |  |
| Occupation (Ref. Blue-collar job) |  |  |  |
| White-collar job | -0.15 | -0.52 to 0.22 | 0.426 |
| Gray-collar job | 0.03 | -0.37 to 0.43 | 0.891 |
| Other/not working | -0.01 | -0.40 to 0.38 | 0.973 |
|  |  |  |  |
| Household annual income (Ref. 7 million yen or more) |  |  |  |
| Less than 3 million yen | 0.33 | 0.01 to 0.65 | 0.044* |
| 3-7 million yen | 0.03 | -0.20 to 0.27 | 0.776 |
| Unknown | -0.03 | -0.36 to 0.31 | 0.879 |
|  |  |  |  |
| Living alone (Ref. No) |  |  |  |
| Yes | 0.01 | -0.27 to 0.29 | 0.950 |
|  |  |  |  |
| Living with child(ren) under 18 years (Ref. No) |  |  |  |
| Yes | 0.12 | -0.13 to 0.36 | 0.353 |
|  |  |  |  |
| Living with person(s) aged 65 years and older (Ref. No) |  |  |  |
| Yes | -0.14 | -0.44 to 0.16 | 0.374 |
|  |  |  |  |
| Neighborhood density (Ref. Lowest density) |  |  |  |
| Middle-low density | 0.16 | -0.10 to 0.42 | 0.238 |
| Middle-high density | -0.01 | -0.29 to 0.26 | 0.919 |
| Highest density | -0.16 | -0.45 to 0.14 | 0.291 |
|  |  |  |  |
| Areal deprivation index (ADI) (Ref. Lowest ADI) |  |  |  |
| Middle-low ADI | -0.29 | -0.56 to -0.03 | 0.031* |
| Middle-high ADI | 0.01 | -0.27 to 0.28 | 0.985 |
| Highest ADI | -0.05 | -0.33 to 0.22 | 0.715 |

Table A.13 (continued)

| From | Coef. | 95% CI | p |
| --- | --- | --- | --- |
| Changes in step counts (in thousands) between the pre-SoE and the post-SoE periods | -0.06 | -0.08 to -0.04 | <0.001*** |
|  |  |  |  |
| Changes in time spent sedentary behavior during the COVID-19 outbreak | 0.11 | 0.04 to 0.19 | 0.004** |

* Coef.: Coefficient and CI: confidence interval. “***,” “**,” and “*,” denote the statistical significance at 0.1%, 1%, and 5% levels, respectively. The sample size was 896.

Table A.14

Estimated coefficients of all paths to strong anxiety about stigma associated with going out for model B

| From | Coef. | 95% CI | p |
| --- | --- | --- | --- |
| Gender (Ref. Male) |  |  |  |
| Female | -0.04 | -0.29 to 0.21 | 0.772 |
|  |  |  |  |
| Age (Ref. 60-69 years) |  |  |  |
| 20-29 years | 0.52 | 0.14 to 0.90 | 0.007** |
| 30-39 years | 0.50 | 0.13 to 0.87 | 0.008** |
| 40-49 years | 0.22 | -0.17 to 0.61 | 0.270 |
| 50-59 years | 0.34 | -0.04 to 0.72 | 0.078 |
|  |  |  |  |
| Chronic disease (Ref. No) |  |  |  |
| Yes | 0.15 | -0.10 to 0.40 | 0.239 |
|  |  |  |  |
| Educational attainment (Ref. Junior high school/high school) |  |  |  |
| Junior (technical) college/vocational school | 0.28 | -0.05 to 0.61 | 0.096 |
| Undergraduate/graduate school | -0.04 | -0.35 to 0.27 | 0.814 |
|  |  |  |  |
| Occupation (Ref. Blue-collar job) |  |  |  |
| White-collar job | -0.09 | -0.48 to 0.30 | 0.660 |
| Gray-collar job | -0.12 | -0.53 to 0.30 | 0.585 |
| Other/not working | -0.01 | -0.42 to 0.41 | 0.979 |
|  |  |  |  |
| Household annual income (Ref. 7 million yen or more) |  |  |  |
| Less than 3 million yen | 0.14 | -0.23 to 0.51 | 0.450 |
| 3-7 million yen | -0.20 | -0.46 to 0.07 | 0.139 |
| Unknown | -0.09 | -0.47 to 0.29 | 0.647 |
|  |  |  |  |
| Living alone (Ref. No) |  |  |  |
| Yes | 0.20 | -0.13 to 0.52 | 0.236 |
|  |  |  |  |
| Living with child(ren) under 18 years (Ref. No) |  |  |  |
| Yes | 0.33 | 0.05 to 0.61 | 0.020* |
|  |  |  |  |
| Living with person(s) aged 65 years and older (Ref. No) |  |  |  |
| Yes | -0.09 | -0.42 to 0.25 | 0.605 |
|  |  |  |  |
| Neighborhood density (Ref. Lowest density) |  |  |  |
| Middle-low density | 0.05 | -0.24 to 0.34 | 0.741 |
| Middle-high density | -0.14 | -0.45 to 0.17 | 0.369 |
| Highest density | -0.36 | -0.69 to -0.04 | 0.030* |
|  |  |  |  |
| Areal deprivation index (ADI) (Ref. Lowest ADI) |  |  |  |
| Middle-low ADI | -0.01 | -0.32 to 0.31 | 0.963 |
| Middle-high ADI | 0.23 | -0.08 to 0.54 | 0.145 |
| Highest ADI | 0.17 | -0.14 to 0.49 | 0.278 |

Table A.14 (continued)

| From | Coef. | 95% CI | p |
| --- | --- | --- | --- |
| Changes in step counts (in thousands) between the pre-SoE and the post-SoE periods | -0.06 | -0.08 to -0.04 | <0.001*** |
|  |  |  |  |
| Changes in time spent sedentary behavior during the COVID-19 outbreak | 0.15 | 0.07 to 0.23 | <0.001*** |

* Coef.: Coefficient and CI: confidence interval. “***,” “**,” and “*,” denote the statistical significance at 0.1%, 1%, and 5% levels, respectively. The sample size was 896.

Table A.15

Estimated coefficients of all paths to introduction of work-from-home/standby-at-home measures for model C

| From | Coef. | 95% CI | p |
| --- | --- | --- | --- |
| Gender (Ref. Male) |  |  |  |
| Female | -0.24 | -0.45 to -0.02 | 0.029* |
|  |  |  |  |
| Age (Ref. 60-69 years) |  |  |  |
| 20-29 years | 0.05 | -0.30 to 0.40 | 0.796 |
| 30-39 years | 0.15 | -0.18 to 0.49 | 0.363 |
| 40-49 years | 0.28 | -0.05 to 0.61 | 0.093 |
| 50-59 years | -0.04 | -0.37 to 0.29 | 0.793 |
|  |  |  |  |
| Chronic disease (Ref. No) |  |  |  |
| Yes | -0.02 | -0.28 to 0.23 | 0.856 |
|  |  |  |  |
| Educational attainment (Ref. Junior high school/high school) |  |  |  |
| Junior (technical) college/vocational school | 0.28 | -0.06 to 0.62 | 0.110 |
| Undergraduate/graduate school | 0.40 | 0.10 to 0.70 | 0.010* |
|  |  |  |  |
| Occupation (Ref. Blue-collar job) |  |  |  |
| White-collar job | 0.59 | 0.20 to 0.97 | 0.003** |
| Gray-collar job | 0.24 | -0.17 to 0.66 | 0.256 |
| Other/not working | -0.77 | -1.24 to -0.30 | 0.001** |
|  |  |  |  |
| Household annual income (Ref. 7 million yen or more) |  |  |  |
| Less than 3 million yen | -0.13 | -0.48 to 0.22 | 0.458 |
| 3-7 million yen | -0.08 | -0.32 to 0.15 | 0.489 |
| Unknown | -0.09 | -0.43 to 0.24 | 0.586 |
|  |  |  |  |
| Living alone (Ref. No) |  |  |  |
| Yes | 0.16 | -0.12 to 0.45 | 0.258 |
|  |  |  |  |
| Living with child(ren) under 18 years (Ref. No) |  |  |  |
| Yes | -0.15 | -0.41 to 0.11 | 0.268 |
|  |  |  |  |
| Living with person(s) aged 65 years and older (Ref. No) |  |  |  |
| Yes | -0.10 | -0.40 to 0.19 | 0.490 |
|  |  |  |  |
| Neighborhood density (Ref. Lowest density) |  |  |  |
| Middle-low density | 0.35 | 0.07 to 0.64 | 0.016* |
| Middle-high density | 0.41 | 0.13 to 0.69 | 0.004** |
| Highest density | 0.64 | 0.34 to 0.94 | <0.001*** |
|  |  |  |  |
| Areal deprivation index (ADI) (Ref. Lowest ADI) |  |  |  |
| Middle-low ADI | 0.02 | -0.26 to 0.29 | 0.901 |
| Middle-high ADI | -0.12 | -0.40 to 0.17 | 0.429 |
| Highest ADI | -0.11 | -0.41 to 0.18 | 0.463 |

* Coef.: Coefficient and CI: confidence interval. “***,” “**,” and “*,” denote the statistical significance at 0.1%, 1%, and 5% levels, respectively. The sample size was 896.

Table A.16

Estimated coefficients of all paths to decreased amount of work for model C

| From | Coef. | 95% CI | p |
| --- | --- | --- | --- |
| Gender (Ref. Male) |  |  |  |
| Female | 0.09 | -0.13 to 0.31 | 0.437 |
|  |  |  |  |
| Age (Ref. 60-69 years) |  |  |  |
| 20-29 years | 0.07 | -0.30 to 0.43 | 0.723 |
| 30-39 years | 0.09 | -0.26 to 0.45 | 0.606 |
| 40-49 years | 0.08 | -0.27 to 0.43 | 0.653 |
| 50-59 years | 0.05 | -0.29 to 0.40 | 0.758 |
|  |  |  |  |
| Chronic disease (Ref. No) |  |  |  |
| Yes | 0.00 | -0.26 to 0.27 | 0.981 |
|  |  |  |  |
| Educational attainment (Ref. Junior high school/high school) |  |  |  |
| Junior (technical) college/vocational school | -0.01 | -0.34 to 0.31 | 0.938 |
| Undergraduate/graduate school | 0.05 | -0.24 to 0.33 | 0.757 |
|  |  |  |  |
| Occupation (Ref. Blue-collar job) |  |  |  |
| White-collar job | -0.18 | -0.56 to 0.19 | 0.335 |
| Gray-collar job | 0.11 | -0.29 to 0.50 | 0.599 |
| Other/not working | -0.68 | -1.09 to -0.27 | 0.001** |
|  |  |  |  |
| Household annual income (Ref. 7 million yen or more) |  |  |  |
| Less than 3 million yen | 0.62 | 0.27 to 0.96 | <0.001*** |
| 3-7 million yen | 0.22 | -0.03 to 0.47 | 0.084 |
| Unknown | 0.08 | -0.31 to 0.48 | 0.685 |
|  |  |  |  |
| Living alone (Ref. No) |  |  |  |
| Yes | 0.11 | -0.18 to 0.40 | 0.449 |
|  |  |  |  |
| Living with child(ren) under 18 years (Ref. No) |  |  |  |
| Yes | 0.03 | -0.24 to 0.31 | 0.813 |
|  |  |  |  |
| Living with person(s) aged 65 years and older (Ref. No) |  |  |  |
| Yes | 0.01 | -0.31 to 0.34 | 0.932 |
|  |  |  |  |
| Neighborhood density (Ref. Lowest density) |  |  |  |
| Middle-low density | -0.17 | -0.47 to 0.13 | 0.274 |
| Middle-high density | -0.03 | -0.32 to 0.27 | 0.859 |
| Highest density | 0.19 | -0.13 to 0.51 | 0.242 |
|  |  |  |  |
| Areal deprivation index (ADI) (Ref. Lowest ADI) |  |  |  |
| Middle-low ADI | 0.29 | -0.02 to 0.59 | 0.066 |
| Middle-high ADI | 0.25 | -0.07 to 0.56 | 0.123 |
| Highest ADI | 0.25 | -0.09 to 0.58 | 0.146 |

* Coef.: Coefficient and CI: confidence interval. “***,” “**,” and “*,” denote the statistical significance at 0.1%, 1%, and 5% levels, respectively. The sample size was 896.

Table A.17

Estimated coefficients of all paths to strong anxiety about getting infected for model C

| From | Coef. | 95% CI | p |
| --- | --- | --- | --- |
| Gender (Ref. Male) |  |  |  |
| Female | 0.40 | 0.13 to 0.67 | 0.003** |
|  |  |  |  |
| Age (Ref. 60-69 years) |  |  |  |
| 20-29 years | -0.05 | -0.52 to 0.42 | 0.837 |
| 30-39 years | 0.24 | -0.19 to 0.67 | 0.276 |
| 40-49 years | 0.05 | -0.37 to 0.46 | 0.820 |
| 50-59 years | 0.27 | -0.16 to 0.70 | 0.213 |
|  |  |  |  |
| Chronic disease (Ref. No) |  |  |  |
| Yes | 0.21 | -0.08 to 0.51 | 0.160 |
|  |  |  |  |
| Educational attainment (Ref. Junior high school/high school) |  |  |  |
| Junior (technical) college/vocational school | -0.03 | -0.43 to 0.37 | 0.889 |
| Undergraduate/graduate school | -0.17 | -0.51 to 0.17 | 0.330 |
|  |  |  |  |
| Occupation (Ref. Blue-collar job) |  |  |  |
| White-collar job | -0.25 | -0.73 to 0.24 | 0.319 |
| Gray-collar job | -0.13 | -0.65 to 0.39 | 0.629 |
| Other/not working | 1.01 | 0.49 to 1.54 | <0.001*** |
|  |  |  |  |
| Household annual income (Ref. 7 million yen or more) |  |  |  |
| Less than 3 million yen | 0.05 | -0.40 to 0.50 | 0.818 |
| 3-7 million yen | -0.02 | -0.33 to 0.30 | 0.917 |
| Unknown | 0.06 | -0.40 to 0.51 | 0.81 |
|  |  |  |  |
| Living alone (Ref. No) |  |  |  |
| Yes | -0.37 | -0.74 to 0.01 | 0.057 |
|  |  |  |  |
| Living with child(ren) under 18 years (Ref. No) |  |  |  |
| Yes | 0.23 | -0.10 to 0.55 | 0.172 |
|  |  |  |  |
| Living with person(s) aged 65 years and older (Ref. No) |  |  |  |
| Yes | -0.08 | -0.46 to 0.30 | 0.684 |
|  |  |  |  |
| Neighborhood density (Ref. Lowest density) |  |  |  |
| Middle-low density | 0.11 | -0.25 to 0.47 | 0.545 |
| Middle-high density | -0.23 | -0.60 to 0.13 | 0.214 |
| Highest density | -0.55 | -0.92 to -0.17 | 0.004** |
|  |  |  |  |
| Areal deprivation index (ADI) (Ref. Lowest ADI) |  |  |  |
| Middle-low ADI | -0.30 | -0.65 to 0.05 | 0.097 |
| Middle-high ADI | -0.12 | -0.48 to 0.24 | 0.512 |
| Highest ADI | -0.12 | -0.50 to 0.26 | 0.545 |

Table A.17 (continued)

| From | Coef. | 95% CI | p |
| --- | --- | --- | --- |
| Introduction of working-from-home/standby at home (Ref. No) |  |  |  |
| Yes | 0.61 | 0.51 to 0.70 | <0.001*** |
|  |  |  |  |
| Decreased amount of work (Ref. No) |  |  |  |
| Yes | 0.72 | 0.63 to 0.80 | <0.001*** |

* Coef.: Coefficient and CI: confidence interval. “***,” “**,” and “*,” denote the statistical significance at 0.1%, 1%, and 5% levels, respectively. The sample size was 896.

Table A.18

Estimated coefficients of all paths to strong anxiety about spreading the infection to others for model C

| From | Coef. | 95% CI | p |
| --- | --- | --- | --- |
| Gender (Ref. Male) |  |  |  |
| Female | 0.38 | 0.11 to 0.65 | 0.006** |
|  |  |  |  |
| Age (Ref. 60-69 years) |  |  |  |
| 20-29 years | 0.06 | -0.40 to 0.52 | 0.815 |
| 30-39 years | 0.26 | -0.17 to 0.69 | 0.239 |
| 40-49 years | -0.03 | -0.47 to 0.40 | 0.880 |
| 50-59 years | 0.14 | -0.31 to 0.58 | 0.548 |
|  |  |  |  |
| Chronic disease (Ref. No) |  |  |  |
| Yes | 0.19 | -0.11 to 0.50 | 0.218 |
|  |  |  |  |
| Educational attainment (Ref. Junior high school/high school) |  |  |  |
| Junior (technical) college/vocational school | -0.03 | -0.44 to 0.38 | 0.881 |
| Undergraduate/graduate school | -0.13 | -0.48 to 0.22 | 0.468 |
|  |  |  |  |
| Occupation (Ref. Blue-collar job) |  |  |  |
| White-collar job | -0.42 | -0.90 to 0.05 | 0.082 |
| Gray-collar job | -0.21 | -0.72 to 0.29 | 0.408 |
| Other/not working | 0.91 | 0.39 to 1.44 | <0.001*** |
|  |  |  |  |
| Household annual income (Ref. 7 million yen or more) |  |  |  |
| Less than 3 million yen | -0.05 | -0.51 to 0.41 | 0.830 |
| 3-7 million yen | -0.06 | -0.38 to 0.26 | 0.710 |
| Unknown | -0.05 | -0.53 to 0.43 | 0.841 |
|  |  |  |  |
| Living alone (Ref. No) |  |  |  |
| Yes | -0.19 | -0.56 to 0.17 | 0.299 |
|  |  |  |  |
| Living with child(ren) under 18 years (Ref. No) |  |  |  |
| Yes | 0.13 | -0.20 to 0.47 | 0.442 |
|  |  |  |  |
| Living with person(s) aged 65 years and older (Ref. No) |  |  |  |
| Yes | -0.10 | -0.49 to 0.29 | 0.602 |
|  |  |  |  |
| Neighborhood density (Ref. Lowest density) |  |  |  |
| Middle-low density | 0.08 | -0.29 to 0.46 | 0.660 |
| Middle-high density | -0.20 | -0.57 to 0.17 | 0.288 |
| Highest density | -0.57 | -0.96 to -0.19 | 0.003** |
|  |  |  |  |
| Areal deprivation index (ADI) (Ref. Lowest ADI) |  |  |  |
| Middle-low ADI | -0.47 | -0.83 to -0.12 | 0.009** |
| Middle-high ADI | -0.12 | -0.49 to 0.25 | 0.535 |
| Highest ADI | -0.18 | -0.57 to 0.22 | 0.379 |

Table A.18 (continued)

| From | Coef. | 95% CI | p |
| --- | --- | --- | --- |
| Introduction of working-from-home/standby at home (Ref. No) |  |  |  |
| Yes | 0.62 | 0.52 to 0.71 | <0.001*** |
|  |  |  |  |
| Decreased amount of work (Ref. No) |  |  |  |
| Yes | 0.70 | 0.62 to 0.79 | <0.001*** |

* Coef.: Coefficient and CI: confidence interval. “***,” “**,” and “*,” denote the statistical significance at 0.1%, 1%, and 5% levels, respectively. The sample size was 896.

Table A.19

Estimated coefficients of all paths to strong anxiety about stigma associated with going out for model C

| From | Coef. | 95% CI | p |
| --- | --- | --- | --- |
| Gender (Ref. Male) |  |  |  |
| Female | 0.09 | -0.21 to 0.38 | 0.568 |
|  |  |  |  |
| Age (Ref. 60-69 years) |  |  |  |
| 20-29 years | 0.59 | 0.11 to 1.08 | 0.017* |
| 30-39 years | 0.40 | -0.06 to 0.87 | 0.085 |
| 40-49 years | 0.09 | -0.39 to 0.56 | 0.719 |
| 50-59 years | 0.34 | -0.12 to 0.81 | 0.151 |
|  |  |  |  |
| Chronic disease (Ref. No) |  |  |  |
| Yes | 0.18 | -0.14 to 0.50 | 0.280 |
|  |  |  |  |
| Educational attainment (Ref. Junior high school/high school) |  |  |  |
| Junior (technical) college/vocational school | 0.16 | -0.27 to 0.58 | 0.469 |
| Undergraduate/graduate school | -0.21 | -0.60 to 0.17 | 0.280 |
|  |  |  |  |
| Occupation (Ref. Blue-collar job) |  |  |  |
| White-collar job | -0.33 | -0.81 to 0.16 | 0.190 |
| Gray-collar job | -0.33 | -0.84 to 0.17 | 0.199 |
| Other/not working | 0.78 | 0.26 to 1.31 | 0.003** |
|  |  |  |  |
| Household annual income (Ref. 7 million yen or more) |  |  |  |
| Less than 3 million yen | -0.21 | -0.69 to 0.28 | 0.401 |
| 3-7 million yen | -0.28 | -0.61 to 0.05 | 0.092 |
| Unknown | -0.12 | -0.62 to 0.37 | 0.626 |
|  |  |  |  |
| Living alone (Ref. No) |  |  |  |
| Yes | 0.01 | -0.38 to 0.41 | 0.949 |
|  |  |  |  |
| Living with child(ren) under 18 years (Ref. No) |  |  |  |
| Yes | 0.33 | -0.01 to 0.67 | 0.058 |
|  |  |  |  |
| Living with person(s) aged 65 years and older (Ref. No) |  |  |  |
| Yes | -0.07 | -0.48 to 0.34 | 0.746 |
|  |  |  |  |
| Neighborhood density (Ref. Lowest density) |  |  |  |
| Middle-low density | 0.00 | -0.38 to 0.38 | 0.993 |
| Middle-high density | -0.28 | -0.66 to 0.09 | 0.140 |
| Highest density | -0.69 | -1.09 to -0.28 | <0.001*** |
|  |  |  |  |
| Areal deprivation index (ADI) (Ref. Lowest ADI) |  |  |  |
| Middle-low ADI | -0.16 | -0.54 to 0.23 | 0.425 |
| Middle-high ADI | 0.11 | -0.28 to 0.50 | 0.564 |
| Highest ADI | 0.06 | -0.35 to 0.46 | 0.783 |

Table A.19 (continued)

| From | Coef. | 95% CI | p |
| --- | --- | --- | --- |
| Introduction of working-from-home/standby at home (Ref. No) |  |  |  |
| Yes | 0.52 | 0.42 to 0.63 | <0.001*** |
|  |  |  |  |
| Decreased amount of work (Ref. No) |  |  |  |
| Yes | 0.62 | 0.53 to 0.71 | <0.001*** |

* Coef.: Coefficient and CI: confidence interval. “***,” “**,” and “*,” denote the statistical significance at 0.1%, 1%, and 5% levels, respectively. The sample size was 896.

Table A.20

Estimated coefficients of all paths to the changes in step counts (in thousands) between the pre-SoE and post-SoE periods for model C

| From | Coef. | 95% CI | p |
| --- | --- | --- | --- |
| Gender (Ref. Male) |  |  |  |
| Female | -0.23 | -0.64 to 0.18 | 0.270 |
|  |  |  |  |
| Age (Ref. 60-69 years) |  |  |  |
| 20-29 years | -1.48 | -2.03 to -0.92 | <0.001*** |
| 30-39 years | -0.47 | -1.12 to 0.17 | 0.151 |
| 40-49 years | -0.65 | -1.24 to -0.05 | 0.033* |
| 50-59 years | -0.42 | -1.02 to 0.18 | 0.170 |
|  |  |  |  |
| Chronic disease (Ref. No) |  |  |  |
| Yes | -0.12 | -0.57 to 0.32 | 0.588 |
|  |  |  |  |
| Educational attainment (Ref. Junior high school/high school) |  |  |  |
| Junior (technical) college/vocational school | 0.09 | -0.52 to 0.70 | 0.777 |
| Undergraduate/graduate school | -0.15 | -0.71 to 0.40 | 0.586 |
|  |  |  |  |
| Occupation (Ref. Blue-collar job) |  |  |  |
| White-collar job | 0.80 | 0.18 to 1.42 | 0.012* |
| Gray-collar job | 0.39 | -0.23 to 1.01 | 0.217 |
| Other/not working | -0.38 | -1.24 to 0.48 | 0.385 |
|  |  |  |  |
| Household annual income (Ref. 7 million yen or more) |  |  |  |
| Less than 3 million yen | 0.07 | -0.49 to 0.63 | 0.801 |
| 3-7 million yen | 0.01 | -0.45 to 0.47 | 0.967 |
| Unknown | -0.14 | -0.73 to 0.46 | 0.656 |
|  |  |  |  |
| Living alone (Ref. No) |  |  |  |
| Yes | 0.48 | -0.03 to 0.98 | 0.063 |
|  |  |  |  |
| Living with child(ren) under 18 years (Ref. No) |  |  |  |
| Yes | 0.49 | -0.02 to 1.01 | 0.061 |
|  |  |  |  |
| Living with person(s) aged 65 years and older (Ref. No) |  |  |  |
| Yes | 0.14 | -0.43 to 0.70 | 0.635 |
|  |  |  |  |
| Neighborhood density (Ref. Lowest density) |  |  |  |
| Middle-low density | -0.01 | -0.50 to 0.48 | 0.966 |
| Middle-high density | -0.32 | -0.84 to 0.20 | 0.223 |
| Highest density | -0.73 | -1.30 to -0.15 | 0.013* |
|  |  |  |  |
| Areal deprivation index (ADI) (Ref. Lowest ADI) |  |  |  |
| Middle-low ADI | -0.16 | -0.66 to 0.34 | 0.526 |
| Middle-high ADI | 0.12 | -0.39 to 0.62 | 0.647 |
| Highest ADI | 0.20 | -0.32 to 0.72 | 0.445 |

Table A.20 (continued)

| From | Coef. | 95% CI | p |
| --- | --- | --- | --- |
| Introduction of working-from-home/standby at home (Ref. No) |  |  |  |
| Yes | -0.65 | -0.99 to -0.31 | <0.001*** |
|  |  |  |  |
| Decreased amount of work (Ref. No) |  |  |  |
| Yes | -0.35 | -0.80 to 0.09 | 0.118 |
|  |  |  |  |
| Strong anxiety about getting infected (Ref. No) |  |  |  |
| Yes | 0.22 | 0.04 to 0.40 | 0.015* |
|  |  |  |  |
| Strong anxiety about spreading the infection to others (Ref. No) |  |  |  |
| Yes | 0.08 | -0.08 to 0.24 | 0.337 |
|  |  |  |  |
| Strong anxiety about stigma associated with going out (Ref. No) |  |  |  |
| Yes | 0.00 | -0.15 to 0.15 | 0.996 |

* Coef.: Coefficient and CI: confidence interval. “***,” “**,” and “*,” denote the statistical significance at 0.1%, 1%, and 5% levels, respectively. The sample size was 896.

Table A.21

Estimated coefficients of all paths to the changes in time spent in sedentary behavior for model C

| From | Coef. | 95% CI | p |
| --- | --- | --- | --- |
| Gender (Ref. Male) |  |  |  |
| Female | 0.57 | 0.32 to 0.82 | <0.001*** |
|  |  |  |  |
| Age (Ref. 60-69 years) |  |  |  |
| 20-29 years | 0.32 | -0.09 to 0.72 | 0.122 |
| 30-39 years | 0.16 | -0.23 to 0.55 | 0.415 |
| 40-49 years | 0.02 | -0.37 to 0.41 | 0.915 |
| 50-59 years | 0.05 | -0.33 to 0.43 | 0.797 |
|  |  |  |  |
| Chronic disease (Ref. No) |  |  |  |
| Yes | 0.19 | -0.08 to 0.47 | 0.171 |
|  |  |  |  |
| Educational attainment (Ref. Junior high school/high school) |  |  |  |
| Junior (technical) college/vocational school | -0.02 | -0.37 to 0.33 | 0.922 |
| Undergraduate/graduate school | 0.04 | -0.29 to 0.37 | 0.814 |
|  |  |  |  |
| Occupation (Ref. Blue-collar job) |  |  |  |
| White-collar job | -0.41 | -0.86 to 0.04 | 0.072 |
| Gray-collar job | -0.26 | -0.72 to 0.19 | 0.251 |
| Other/not working | 0.77 | 0.18 to 1.36 | 0.011* |
|  |  |  |  |
| Household annual income (Ref. 7 million yen or more) |  |  |  |
| Less than 3 million yen | -0.32 | -0.71 to 0.06 | 0.098 |
| 3-7 million yen | 0.01 | -0.27 to 0.29 | 0.941 |
| Unknown | -0.25 | -0.66 to 0.15 | 0.226 |
|  |  |  |  |
| Living alone (Ref. No) |  |  |  |
| Yes | -0.25 | -0.57 to 0.07 | 0.119 |
|  |  |  |  |
| Living with child(ren) under 18 years (Ref. No) |  |  |  |
| Yes | 0.01 | -0.30 to 0.31 | 0.961 |
|  |  |  |  |
| Living with person(s) aged 65 years and older (Ref. No) |  |  |  |
| Yes | -0.12 | -0.44 to 0.19 | 0.442 |
|  |  |  |  |
| Neighborhood density (Ref. Lowest density) |  |  |  |
| Middle-low density | 0.14 | -0.19 to 0.47 | 0.414 |
| Middle-high density | -0.10 | -0.43 to 0.23 | 0.564 |
| Highest density | -0.17 | -0.57 to 0.23 | 0.400 |
|  |  |  |  |
| Areal deprivation index (ADI) (Ref. Lowest ADI) |  |  |  |
| Middle-low ADI | -0.11 | -0.44 to 0.21 | 0.499 |
| Middle-high ADI | -0.16 | -0.47 to 0.15 | 0.320 |
| Highest ADI | -0.15 | -0.47 to 0.17 | 0.357 |

Table A.21 (continued)

| From | Coef. | 95% CI | p |
| --- | --- | --- | --- |
| Introduction of working-from-home/standby at home (Ref. No) |  |  |  |
| Yes | 0.60 | 0.36 to 0.85 | <0.001*** |
|  |  |  |  |
| Decreased amount of work (Ref. No) |  |  |  |
| Yes | 0.60 | 0.31 to 0.88 | <0.001*** |
|  |  |  |  |
| Strong anxiety about getting infected (Ref. No) |  |  |  |
| Yes | -0.20 | -0.32 to -0.09 | <0.001*** |
|  |  |  |  |
| Strong anxiety about spreading the infection to others (Ref. No) |  |  |  |
| Yes | -0.21 | -0.32 to -0.09 | <0.001*** |
|  |  |  |  |
| Strong anxiety about stigma associated with going out (Ref. No) |  |  |  |
| Yes | -0.10 | -0.22 to 0.03 | 0.128 |

* Coef.: Coefficient and CI: confidence interval. “***,” “**,” and “*,” denote the statistical significance at 0.1%, 1%, and 5% levels, respectively. The sample size was 896. The ordered categories of change in time spent in sedentary behavior were defined as follows: 1: significant reduction, 2: slight reduction, 3: no change, 4: slight rise, 5: significant rise.

Table A.22

Estimated coefficients of all paths to introduction of work-from-home/standby-at-home measures for model D

| From | Coef. | 95% CI | p |
| --- | --- | --- | --- |
| Gender (Ref. Male) |  |  |  |
| Female | -0.24 | -0.45 to -0.02 | 0.029* |
|  |  |  |  |
| Age (Ref. 60-69 years) |  |  |  |
| 20-29 years | 0.05 | -0.30 to 0.40 | 0.796 |
| 30-39 years | 0.15 | -0.18 to 0.49 | 0.363 |
| 40-49 years | 0.28 | -0.05 to 0.61 | 0.093 |
| 50-59 years | -0.04 | -0.37 to 0.29 | 0.793 |
|  |  |  |  |
| Chronic disease (Ref. No) |  |  |  |
| Yes | -0.02 | -0.28 to 0.23 | 0.856 |
|  |  |  |  |
| Educational attainment (Ref. Junior high school/high school) |  |  |  |
| Junior (technical) college/vocational school | 0.28 | -0.06 to 0.62 | 0.110 |
| Undergraduate/graduate school | 0.40 | 0.10 to 0.70 | 0.010* |
|  |  |  |  |
| Occupation (Ref. Blue-collar job) |  |  |  |
| White-collar job | 0.59 | 0.20 to 0.97 | 0.003** |
| Gray-collar job | 0.24 | -0.17 to 0.66 | 0.256 |
| Other/not working | -0.77 | -1.24 to -0.30 | 0.001** |
|  |  |  |  |
| Household annual income (Ref. 7 million yen or more) |  |  |  |
| Less than 3 million yen | -0.13 | -0.48 to 0.22 | 0.458 |
| 3-7 million yen | -0.08 | -0.32 to 0.15 | 0.489 |
| Unknown | -0.09 | -0.43 to 0.24 | 0.586 |
|  |  |  |  |
| Living alone (Ref. No) |  |  |  |
| Yes | 0.16 | -0.12 to 0.45 | 0.257 |
|  |  |  |  |
| Living with child(ren) under 18 years (Ref. No) |  |  |  |
| Yes | -0.15 | -0.41 to 0.11 | 0.268 |
|  |  |  |  |
| Living with person(s) aged 65 years and older (Ref. No) |  |  |  |
| Yes | -0.10 | -0.40 to 0.19 | 0.490 |
|  |  |  |  |
| Neighborhood density (Ref. Lowest density) |  |  |  |
| Middle-low density | 0.35 | 0.07 to 0.64 | 0.016* |
| Middle-high density | 0.41 | 0.13 to 0.69 | 0.004** |
| Highest density | 0.64 | 0.34 to 0.94 | <0.001*** |
|  |  |  |  |
| Areal deprivation index (ADI) (Ref. Lowest ADI) |  |  |  |
| Middle-low ADI | 0.02 | -0.26 to 0.29 | 0.901 |
| Middle-high ADI | -0.12 | -0.40 to 0.17 | 0.429 |
| Highest ADI | -0.11 | -0.41 to 0.18 | 0.463 |

* Coef.: Coefficient and CI: confidence interval. “***,” “**,” and “*,” denote the statistical significance at 0.1%, 1%, and 5% levels, respectively. The sample size was 896.

Table A.23

Estimated coefficients of all paths to decreased amount of work for model D

| From | Coef. | 95% CI | p |
| --- | --- | --- | --- |
| Gender (Ref. Male) |  |  |  |
| Female | 0.09 | -0.13 to 0.31 | 0.437 |
|  |  |  |  |
| Age (Ref. 60-69 years) |  |  |  |
| 20-29 years | 0.07 | -0.30 to 0.43 | 0.723 |
| 30-39 years | 0.09 | -0.26 to 0.45 | 0.606 |
| 40-49 years | 0.08 | -0.27 to 0.43 | 0.653 |
| 50-59 years | 0.05 | -0.29 to 0.40 | 0.758 |
|  |  |  |  |
| Chronic disease (Ref. No) |  |  |  |
| Yes | 0.00 | -0.26 to 0.27 | 0.981 |
|  |  |  |  |
| Educational attainment (Ref. Junior high school/high school) |  |  |  |
| Junior (technical) college/vocational school | -0.01 | -0.34 to 0.31 | 0.938 |
| Undergraduate/graduate school | 0.05 | -0.24 to 0.33 | 0.757 |
|  |  |  |  |
| Occupation (Ref. Blue-collar job) |  |  |  |
| White-collar job | -0.18 | -0.56 to 0.19 | 0.336 |
| Gray-collar job | 0.11 | -0.29 to 0.50 | 0.599 |
| Other/not working | -0.68 | -1.09 to -0.27 | 0.001** |
|  |  |  |  |
| Household annual income (Ref. 7 million yen or more) |  |  |  |
| Less than 3 million yen | 0.62 | 0.27 to 0.96 | <0.001*** |
| 3-7 million yen | 0.22 | -0.03 to 0.47 | 0.084 |
| Unknown | 0.08 | -0.31 to 0.48 | 0.685 |
|  |  |  |  |
| Living alone (Ref. No) |  |  |  |
| Yes | 0.11 | -0.18 to 0.40 | 0.449 |
|  |  |  |  |
| Living with child(ren) under 18 years (Ref. No) |  |  |  |
| Yes | 0.03 | -0.24 to 0.31 | 0.813 |
|  |  |  |  |
| Living with person(s) aged 65 years and older (Ref. No) |  |  |  |
| Yes | 0.01 | -0.31 to 0.34 | 0.932 |
|  |  |  |  |
| Neighborhood density (Ref. Lowest density) |  |  |  |
| Middle-low density | -0.17 | -0.47 to 0.13 | 0.274 |
| Middle-high density | -0.03 | -0.32 to 0.27 | 0.859 |
| Highest density | 0.19 | -0.13 to 0.51 | 0.242 |
|  |  |  |  |
| Areal deprivation index (ADI) (Ref. Lowest ADI) |  |  |  |
| Middle-low ADI | 0.29 | -0.02 to 0.59 | 0.066 |
| Middle-high ADI | 0.25 | -0.07 to 0.56 | 0.123 |
| Highest ADI | 0.25 | -0.09 to 0.58 | 0.146 |

* Coef.: Coefficient and CI: confidence interval. “***,” “**,” and “*,” denote the statistical significance at 0.1%, 1%, and 5% levels, respectively. The sample size was 896.

Table A.24

Estimated coefficients of all paths to the changes in step counts (in thousands) between the pre-SoE and post-SoE periods for model D

| From | Coef. | 95% CI | p |
| --- | --- | --- | --- |
| Gender (Ref. Male) |  |  |  |
| Female | -0.13 | -0.50 to 0.25 | 0.513 |
|  |  |  |  |
| Age (Ref. 60-69 years) |  |  |  |
| 20-29 years | -1.47 | -2.00 to -0.94 | <0.001*** |
| 30-39 years | -0.37 | -0.99 to 0.25 | 0.246 |
| 40-49 years | -0.59 | -1.17 to -0.02 | 0.043* |
| 50-59 years | -0.35 | -0.92 to 0.22 | 0.233 |
|  |  |  |  |
| Chronic disease (Ref. No) |  |  |  |
| Yes | -0.06 | -0.49 to 0.36 | 0.773 |
|  |  |  |  |
| Educational attainment (Ref. Junior high school/high school) |  |  |  |
| Junior (technical) college/vocational school | 0.11 | -0.49 to 0.71 | 0.717 |
| Undergraduate/graduate school | -0.15 | -0.68 to 0.39 | 0.594 |
|  |  |  |  |
| Occupation (Ref. Blue-collar job) |  |  |  |
| White-collar job | 0.76 | 0.17 to 1.34 | 0.011* |
| Gray-collar job | 0.39 | -0.20 to 0.98 | 0.195 |
| Other/not working | -0.28 | -0.93 to 0.37 | 0.403 |
|  |  |  |  |
| Household annual income (Ref. 7 million yen or more) |  |  |  |
| Less than 3 million yen | 0.16 | -0.39 to 0.70 | 0.570 |
| 3-7 million yen | 0.02 | -0.42 to 0.47 | 0.914 |
| Unknown | -0.13 | -0.70 to 0.45 | 0.669 |
|  |  |  |  |
| Living alone (Ref. No) |  |  |  |
| Yes | 0.42 | -0.06 to 0.89 | 0.086 |
|  |  |  |  |
| Living with child(ren) under 18 years (Ref. No) |  |  |  |
| Yes | 0.54 | 0.05 to 1.03 | 0.030* |
|  |  |  |  |
| Living with person(s) aged 65 years and older (Ref. No) |  |  |  |
| Yes | 0.10 | -0.45 to 0.65 | 0.720 |
|  |  |  |  |
| Neighborhood density (Ref. Lowest density) |  |  |  |
| Middle-low density | 0.04 | -0.44 to 0.51 | 0.875 |
| Middle-high density | -0.34 | -0.82 to 0.14 | 0.160 |
| Highest density | -0.79 | -1.25 to -0.32 | <0.001*** |
|  |  |  |  |
| Areal deprivation index (ADI) (Ref. Lowest ADI) |  |  |  |
| Middle-low ADI | -0.22 | -0.68 to 0.24 | 0.347 |
| Middle-high ADI | 0.10 | -0.38 to 0.59 | 0.674 |
| Highest ADI | 0.19 | -0.32 to 0.69 | 0.467 |

Table A.24 (continued)

| From | Coef. | 95% CI | p |
| --- | --- | --- | --- |
| Introduction of working-from-home/standby at home (Ref. No) |  |  |  |
| Yes | -0.59 | -0.71 to -0.46 | <0.001*** |
|  |  |  |  |
| Decreased amount of work (Ref. No) |  |  |  |
| Yes | -0.29 | -0.45 to -0.13 | <0.001*** |

* Coef.: Coefficient and CI: confidence interval. “***,” “**,” and “*,” denote the statistical significance at 0.1%, 1%, and 5% levels, respectively. The sample size was 896.

Table A.25

Estimated coefficients of all paths to the changes in time spent in sedentary behavior for model D

| From | Coef. | 95% CI | p |
| --- | --- | --- | --- |
| Gender (Ref. Male) |  |  |  |
| Female | 0.42 | 0.24 to 0.59 | <0.001*** |
|  |  |  |  |
| Age (Ref. 60-69 years) |  |  |  |
| 20-29 years | 0.25 | -0.02 to 0.53 | 0.074 |
| 30-39 years | 0.01 | -0.27 to 0.28 | 0.967 |
| 40-49 years | -0.02 | -0.30 to 0.27 | 0.917 |
| 50-59 years | -0.07 | -0.33 to 0.20 | 0.632 |
|  |  |  |  |
| Chronic disease (Ref. No) |  |  |  |
| Yes | 0.09 | -0.10 to 0.29 | 0.347 |
|  |  |  |  |
| Educational attainment (Ref. Junior high school/high school) |  |  |  |
| Junior (technical) college/vocational school | -0.04 | -0.29 to 0.21 | 0.751 |
| Undergraduate/graduate school | 0.09 | -0.15 to 0.33 | 0.470 |
|  |  |  |  |
| Occupation (Ref. Blue-collar job) |  |  |  |
| White-collar job | -0.28 | -0.61 to 0.05 | 0.094 |
| Gray-collar job | -0.19 | -0.52 to 0.15 | 0.282 |
| Other/not working | 0.39 | 0.03 to 0.75 | 0.034* |
|  |  |  |  |
| Household annual income (Ref. 7 million yen or more) |  |  |  |
| Less than 3 million yen | -0.32 | -0.60 to -0.05 | 0.021* |
| 3-7 million yen | 0.05 | -0.15 to 0.25 | 0.630 |
| Unknown | -0.24 | -0.53 to 0.05 | 0.107 |
|  |  |  |  |
| Living alone (Ref. No) |  |  |  |
| Yes | -0.16 | -0.39 to 0.07 | 0.183 |
|  |  |  |  |
| Living with child(ren) under 18 years (Ref. No) |  |  |  |
| Yes | -0.09 | -0.31 to 0.13 | 0.433 |
|  |  |  |  |
| Living with person(s) aged 65 years and older (Ref. No) |  |  |  |
| Yes | -0.07 | -0.30 to 0.15 | 0.523 |
|  |  |  |  |
| Neighborhood density (Ref. Lowest density) |  |  |  |
| Middle-low density | 0.08 | -0.16 to 0.32 | 0.518 |
| Middle-high density | -0.01 | -0.25 to 0.23 | 0.926 |
| Highest density | 0.07 | -0.17 to 0.30 | 0.587 |
|  |  |  |  |
| Areal deprivation index (ADI) (Ref. Lowest ADI) |  |  |  |
| Middle-low ADI | 0.05 | -0.18 to 0.27 | 0.699 |
| Middle-high ADI | -0.12 | -0.35 to 0.10 | 0.287 |
| Highest ADI | -0.10 | -0.34 to 0.13 | 0.400 |

Table A.25 (continued)

| From | Coef. | 95% CI | p |
| --- | --- | --- | --- |
| Introduction of working-from-home/standby at home (Ref. No) |  |  |  |
| Yes | 0.38 | 0.30 to 0.46 | <0.001*** |
|  |  |  |  |
| Decreased amount of work (Ref. No) |  |  |  |
| Yes | 0.29 | 0.20 to 0.39 | <0.001*** |

* Coef.: Coefficient and CI: confidence interval. “***,” “**,” and “*,” denote the statistical significance at 0.1%, 1%, and 5% levels, respectively. The sample size was 896. The ordered categories of change in time spent in sedentary behavior were defined as follows: 1: significant reduction, 2: slight reduction, 3: no change, 4: slight rise, 5: significant rise.

Table A.26

Estimated coefficients of all paths to strong anxiety about getting infected for model D

| From | Coef. | 95% CI | p |
| --- | --- | --- | --- |
| Gender (Ref. Male) |  |  |  |
| Female | 0.29 | 0.08 to 0.49 | 0.006** |
|  |  |  |  |
| Age (Ref. 60-69 years) |  |  |  |
| 20-29 years | -0.04 | -0.37 to 0.29 | 0.814 |
| 30-39 years | 0.37 | 0.07 to 0.67 | 0.015* |
| 40-49 years | 0.24 | -0.06 to 0.53 | 0.114 |
| 50-59 years | 0.28 | -0.01 to 0.57 | 0.059 |
|  |  |  |  |
| Chronic disease (Ref. No) |  |  |  |
| Yes | 0.19 | -0.02 to 0.40 | 0.074 |
|  |  |  |  |
| Educational attainment (Ref. Junior high school/high school) |  |  |  |
| Junior (technical) college/vocational school | 0.12 | -0.16 to 0.40 | 0.408 |
| Undergraduate/graduate school | 0.06 | -0.19 to 0.31 | 0.628 |
|  |  |  |  |
| Occupation (Ref. Blue-collar job) |  |  |  |
| White-collar job | -0.01 | -0.37 to 0.36 | 0.969 |
| Gray-collar job | 0.09 | -0.30 to 0.48 | 0.642 |
| Other/not working | 0.13 | -0.28 to 0.53 | 0.533 |
|  |  |  |  |
| Household annual income (Ref. 7 million yen or more) |  |  |  |
| Less than 3 million yen | 0.41 | 0.08 to 0.74 | 0.015* |
| 3-7 million yen | 0.07 | -0.15 to 0.29 | 0.516 |
| Unknown | 0.08 | -0.24 to 0.40 | 0.630 |
|  |  |  |  |
| Living alone (Ref. No) |  |  |  |
| Yes | -0.18 | -0.46 to 0.10 | 0.204 |
|  |  |  |  |
| Living with child(ren) under 18 years (Ref. No) |  |  |  |
| Yes | 0.19 | -0.05 to 0.42 | 0.117 |
|  |  |  |  |
| Living with person(s) aged 65 years and older (Ref. No) |  |  |  |
| Yes | -0.12 | -0.39 to 0.15 | 0.401 |
|  |  |  |  |
| Neighborhood density (Ref. Lowest density) |  |  |  |
| Middle-low density | 0.19 | -0.07 to 0.44 | 0.151 |
| Middle-high density | -0.04 | -0.30 to 0.22 | 0.783 |
| Highest density | -0.11 | -0.39 to 0.17 | 0.438 |
|  |  |  |  |
| Areal deprivation index (ADI) (Ref. Lowest ADI) |  |  |  |
| Middle-low ADI | -0.11 | -0.37 to 0.14 | 0.388 |
| Middle-high ADI | -0.01 | -0.28 to 0.25 | 0.930 |
| Highest ADI | -0.01 | -0.27 to 0.26 | 0.961 |

Table A.26 (continued)

| From | Coef. | 95% CI | p |
| --- | --- | --- | --- |
| Introduction of working-from-home/standby at home (Ref. No) |  |  |  |
| Yes | 0.03 | -0.12 to 0.17 | 0.735 |
|  |  |  |  |
| Decreased amount of work (Ref. No) |  |  |  |
| Yes | 0.05 | -0.08 to 0.18 | 0.447 |
|  |  |  |  |
| Changes in step counts (in thousands) between the pre-SoE and the post-SoE periods | -0.02 | -0.06 to 0.01 | 0.224 |
|  |  |  |  |
| Changes in time spent sedentary behavior during the COVID-19 outbreak | 0.10 | 0.01 to 0.19 | 0.035* |

* Coef.: Coefficient and CI: confidence interval. “***,” “**,” and “*,” denote the statistical significance at 0.1%, 1%, and 5% levels, respectively. The sample size was 896.

Table A.27

Estimated coefficients of all paths to strong anxiety about spreading the infection to others for model D

| From | Coef. | 95% CI | p |
| --- | --- | --- | --- |
| Gender (Ref. Male) |  |  |  |
| Female | 0.25 | 0.04 to 0.47 | 0.020* |
|  |  |  |  |
| Age (Ref. 60-69 years) |  |  |  |
| 20-29 years | 0.01 | -0.33 to 0.35 | 0.954 |
| 30-39 years | 0.38 | 0.07 to 0.69 | 0.018* |
| 40-49 years | 0.14 | -0.18 to 0.45 | 0.393 |
| 50-59 years | 0.13 | -0.19 to 0.45 | 0.424 |
|  |  |  |  |
| Chronic disease (Ref. No) |  |  |  |
| Yes | 0.17 | -0.05 to 0.39 | 0.136 |
|  |  |  |  |
| Educational attainment (Ref. Junior high school/high school) |  |  |  |
| Junior (technical) college/vocational school | 0.13 | -0.17 to 0.42 | 0.398 |
| Undergraduate/graduate school | 0.10 | -0.16 to 0.37 | 0.450 |
|  |  |  |  |
| Occupation (Ref. Blue-collar job) |  |  |  |
| White-collar job | -0.14 | -0.51 to 0.24 | 0.477 |
| Gray-collar job | 0.02 | -0.38 to 0.42 | 0.907 |
| Other/not working | 0.02 | -0.40 to 0.43 | 0.930 |
|  |  |  |  |
| Household annual income (Ref. 7 million yen or more) |  |  |  |
| Less than 3 million yen | 0.29 | -0.04 to 0.63 | 0.083 |
| 3-7 million yen | 0.02 | -0.21 to 0.26 | 0.849 |
| Unknown | -0.03 | -0.37 to 0.30 | 0.844 |
|  |  |  |  |
| Living alone (Ref. No) |  |  |  |
| Yes | 0.00 | -0.28 to 0.29 | 0.979 |
|  |  |  |  |
| Living with child(ren) under 18 years (Ref. No) |  |  |  |
| Yes | 0.11 | -0.14 to 0.36 | 0.376 |
|  |  |  |  |
| Living with person(s) aged 65 years and older (Ref. No) |  |  |  |
| Yes | -0.14 | -0.44 to 0.16 | 0.360 |
|  |  |  |  |
| Neighborhood density (Ref. Lowest density) |  |  |  |
| Middle-low density | 0.17 | -0.10 to 0.44 | 0.207 |
| Middle-high density | -0.01 | -0.28 to 0.27 | 0.963 |
| Highest density | -0.16 | -0.46 to 0.14 | 0.310 |
|  |  |  |  |
| Areal deprivation index (ADI) (Ref. Lowest ADI) |  |  |  |
| Middle-low ADI | -0.31 | -0.57 to -0.04 | 0.025* |
| Middle-high ADI | -0.01 | -0.29 to 0.26 | 0.931 |
| Highest ADI | -0.07 | -0.34 to 0.21 | 0.640 |

Table A.27 (continued)

| From | Coef. | 95% CI | p |
| --- | --- | --- | --- |
| Introduction of working-from-home/standby at home (Ref. No) |  |  |  |
| Yes | -0.01 | -0.16 to 0.14 | 0.901 |
|  |  |  |  |
| Decreased amount of work (Ref. No) |  |  |  |
| Yes | 0.05 | -0.08 to 0.18 | 0.457 |
|  |  |  |  |
| Changes in step counts (in thousands) between the pre-SoE and the post-SoE periods | -0.06 | -0.09 to -0.03 | <0.001*** |
|  |  |  |  |
| Changes in time spent sedentary behavior during the COVID-19 outbreak | 0.10 | 0.01 to 0.19 | 0.035* |

* Coef.: Coefficient and CI: confidence interval. “***,” “**,” and “*,” denote the statistical significance at 0.1%, 1%, and 5% levels, respectively. The sample size was 896.

Table A.28

Estimated coefficients of all paths to strong anxiety about stigma associated with going out for model D

| From | Coef. | 95% CI | p |
| --- | --- | --- | --- |
| Gender (Ref. Male) |  |  |  |
| Female | -0.07 | -0.33 to 0.19 | 0.599 |
|  |  |  |  |
| Age (Ref. 60-69 years) |  |  |  |
| 20-29 years | 0.50 | 0.12 to 0.89 | 0.011* |
| 30-39 years | 0.51 | 0.14 to 0.87 | 0.007** |
| 40-49 years | 0.23 | -0.16 to 0.62 | 0.244 |
| 50-59 years | 0.34 | -0.04 to 0.72 | 0.083 |
|  |  |  |  |
| Chronic disease (Ref. No) |  |  |  |
| Yes | 0.14 | -0.10 to 0.39 | 0.255 |
|  |  |  |  |
| Educational attainment (Ref. Junior high school/high school) |  |  |  |
| Junior (technical) college/vocational school | 0.30 | -0.03 to 0.64 | 0.076 |
| Undergraduate/graduate school | -0.01 | -0.33 to 0.30 | 0.929 |
|  |  |  |  |
| Occupation (Ref. Blue-collar job) |  |  |  |
| White-collar job | -0.03 | -0.43 to 0.37 | 0.887 |
| Gray-collar job | -0.09 | -0.51 to 0.33 | 0.673 |
| Other/not working | -0.07 | -0.52 to 0.39 | 0.765 |
|  |  |  |  |
| Household annual income (Ref. 7 million yen or more) |  |  |  |
| Less than 3 million yen | 0.14 | -0.24 to 0.52 | 0.469 |
| 3-7 million yen | -0.21 | -0.48 to 0.06 | 0.127 |
| Unknown | -0.09 | -0.47 to 0.29 | 0.644 |
|  |  |  |  |
| Living alone (Ref. No) |  |  |  |
| Yes | 0.22 | -0.11 to 0.54 | 0.199 |
|  |  |  |  |
| Living with child(ren) under 18 years (Ref. No) |  |  |  |
| Yes | 0.33 | 0.05 to 0.61 | 0.021* |
|  |  |  |  |
| Living with person(s) aged 65 years and older (Ref. No) |  |  |  |
| Yes | -0.09 | -0.43 to 0.24 | 0.591 |
|  |  |  |  |
| Neighborhood density (Ref. Lowest density) |  |  |  |
| Middle-low density | 0.07 | -0.22 to 0.37 | 0.631 |
| Middle-high density | -0.11 | -0.42 to 0.20 | 0.469 |
| Highest density | -0.33 | -0.66 to 0.01 | 0.054 |
|  |  |  |  |
| Areal deprivation index (ADI) (Ref. Lowest ADI) |  |  |  |
| Middle-low ADI | -0.01 | -0.33 to 0.31 | 0.941 |
| Middle-high ADI | 0.22 | -0.09 to 0.54 | 0.158 |
| Highest ADI | 0.17 | -0.15 to 0.49 | 0.292 |

Table A.28 (continued)

| From | Coef. | 95% CI | p |
| --- | --- | --- | --- |
| Introduction of working-from-home/standby at home (Ref. No) |  |  |  |
| Yes | -0.09 | -0.25 to 0.08 | 0.298 |
|  |  |  |  |
| Decreased amount of work (Ref. No) |  |  |  |
| Yes | -0.01 | -0.15 to 0.14 | 0.942 |
|  |  |  |  |
| Changes in step counts (in thousands) between the pre-SoE and the post-SoE periods | -0.07 | -0.10 to -0.04 | <0.001*** |
|  |  |  |  |
| Changes in time spent sedentary behavior during the COVID-19 outbreak | 0.18 | 0.08 to 0.29 | <0.001*** |

* Coef.: Coefficient and CI: confidence interval. “***,” “**,” and “*,” denote the statistical significance at 0.1%, 1%, and 5% levels, respectively. The sample size was 896.
